# Calcium Regulation of Hemorrhagic Fever Virus Budding: Mechanistic Implications for Host-Oriented Therapeutic Intervention

**DOI:** 10.1371/journal.ppat.1005220

**Published:** 2015-10-29

**Authors:** Ziying Han, Jonathan J. Madara, Andrew Herbert, Laura I. Prugar, Gordon Ruthel, Jianhong Lu, Yuliang Liu, Wenbo Liu, Xiaohong Liu, Jay E. Wrobel, Allen B. Reitz, John M. Dye, Ronald N. Harty, Bruce D. Freedman

**Affiliations:** 1 Department of Pathobiology, School of Veterinary Medicine, University of Pennsylvania, Philadelphia, Pennsylvania, United States of America; 2 United States Army Medical Research Institute of Infectious Diseases, Fort Detrick, Maryland, United States of America; 3 Fox Chase Chemical Diversity Center, Inc., Doylestown, Pennsylvania, United States of America; Integrated Research Facility at Fort Detrick, UNITED STATES

## Abstract

Hemorrhagic fever viruses, including the filoviruses (Ebola and Marburg) and arenaviruses (Lassa and Junín viruses), are serious human pathogens for which there are currently no FDA approved therapeutics or vaccines. Importantly, transmission of these viruses, and specifically late steps of budding, critically depend upon host cell machinery. Consequently, strategies which target these mechanisms represent potential targets for broad spectrum host oriented therapeutics. An important cellular signal implicated previously in EBOV budding is calcium. Indeed, host cell calcium signals are increasingly being recognized to play a role in steps of entry, replication, and transmission for a range of viruses, but if and how filoviruses and arenaviruses mobilize calcium and the precise stage of virus transmission regulated by calcium have not been defined. Here we demonstrate that expression of matrix proteins from both filoviruses and arenaviruses triggers an increase in host cytoplasmic Ca^2+^ concentration by a mechanism that requires host Orai1 channels. Furthermore, we demonstrate that Orai1 regulates both VLP and infectious filovirus and arenavirus production and spread. Notably, suppression of the protein that triggers Orai activation (Stromal Interaction Molecule 1, STIM1) and genetic inactivation or pharmacological blockade of Orai1 channels inhibits VLP and infectious virus egress. These findings are highly significant as they expand our understanding of host mechanisms that may broadly control enveloped RNA virus budding, and they establish Orai and STIM1 as novel targets for broad-spectrum host-oriented therapeutics to combat these emerging BSL-4 pathogens and potentially other enveloped RNA viruses that bud via similar mechanisms.

## Author Summary

Filoviruses (Ebola and Marburg viruses) and arenaviruses (Lassa and Junín viruses) are high-priority pathogens that hijack host proteins and pathways to complete their replication cycles and spread from cell to cell. Here we provide genetic and pharmacological evidence to demonstrate that the host calcium channel protein Orai1 and ER calcium sensor protein STIM1 regulate efficient budding and spread of BSL-4 pathogens Ebola, Marburg, Lassa, and Junín viruses. Our findings are of broad significance as they provide new mechanistic insight into fundamental, immutable, and conserved mechanisms of hemorrhagic fever virus pathogenesis. Moreover, this strategy of targeting highly conserved host cellular protein(s) and mechanisms required by these viruses to complete their life cycle should elicit minimal drug resistance.

## Introduction

There is an urgent and unmet need for safe and effective therapeutics against high priority pathogens, including filoviruses (Ebola and Marburg) and arenaviruses (Lassa fever and Junín), which can cause fatal infections in humans. We and others have established that enveloped RNA viruses, including hemorrhagic fever viruses, exhibit a common requirement for host pathways, most notably ESCRT pathway functions, for efficient budding [1–7]. Indeed as host dependent budding mechanisms are highly conserved within and sometimes across virus families, they represent innovative and immutable antiviral targets for inhibiting virus transmission and disease progression [8–11]. Importantly, high mutation rates of RNA viruses in general are a factor in their ability to develop resistance to therapeutics that target specific viral proteins or functions [3, 12–23]. Consequently, strategies that target specific host mechanisms required by viruses should reduce the development of resistance.

As a number of these host mechanisms, including steps in ESCRT protein function, are targets of calcium regulation, the focus of this study was to determine whether and how hemorrhagic fever viruses mobilize calcium in host cells and whether calcium so mobilized regulates virus budding. Here we reveal a novel and fundamental requirement for host STIM1- and Orai-mediated Ca^2+^ entry that regulates late steps of filovirus and arenavirus egress from mammalian cells. Orai activation is typically linked to either tyrosine kinase or G-protein coupled receptors that activate phospholipase C (PLC) and generate diacylglycerol and inositol 1,4,5-triphoshate (IP3) from membrane phospholipids. IP3 activates receptor/channels on the endoplasmic reticulum (ER) to allow Ca^2+^ to exit from the ER. The subsequent drop in ER Ca^2+^ below the K_D_ (400–600μM, [24]) for the N-terminal EF hands of the ER membrane-resident protein STIM1 initiates a conformational change that promotes STIM1 oligomerization and localization to ER regions adjacent to the plasma membrane. At the plasma membrane, STIM1 interacts with and activates Calcium-Release Activated Calcium (CRAC) channels through which extracellular Ca^2+^ enters the cell (reviewed in [25]). CRAC channels are encoded by the Orai family of proteins (Orai1, 2, & 3; [26–28]) that provide a pathway for sustained extracellular Ca^2+^ entry to regulate a range of cell functions including gene expression, subcellular trafficking, and the regulation of cell shape and motility [29–31].

Herein, we demonstrate that both filovirus (VP40) and arenavirus (Z) matrix proteins trigger Orai dependent Ca^2+^ entry in mammalian cells. In addition, suppression of STIM1 expression and genetic inactivation or pharmacological blockade of Orai inhibits Ebolavirus (EBOV), Marburgvirus (MARV), Lassa Virus (LASV), and Junín Virus (JUNV) VLP and infectious virion production and transmission in cell culture. Together, these data establish a novel and critical role for STIM1- and Orai-mediated Ca^2+^ entry in late steps of hemorrhagic fever virus egress and establish STIM1 and Orai inhibitors as potential broad-spectrum anti-viral targets for regulation of these and possibly other enveloped RNA viruses that bud by similar mechanisms.

## Results

### Viral VP40 and Z matrix protein expression mobilizes cytoplasmic Ca^2+^

**Fig 1:**
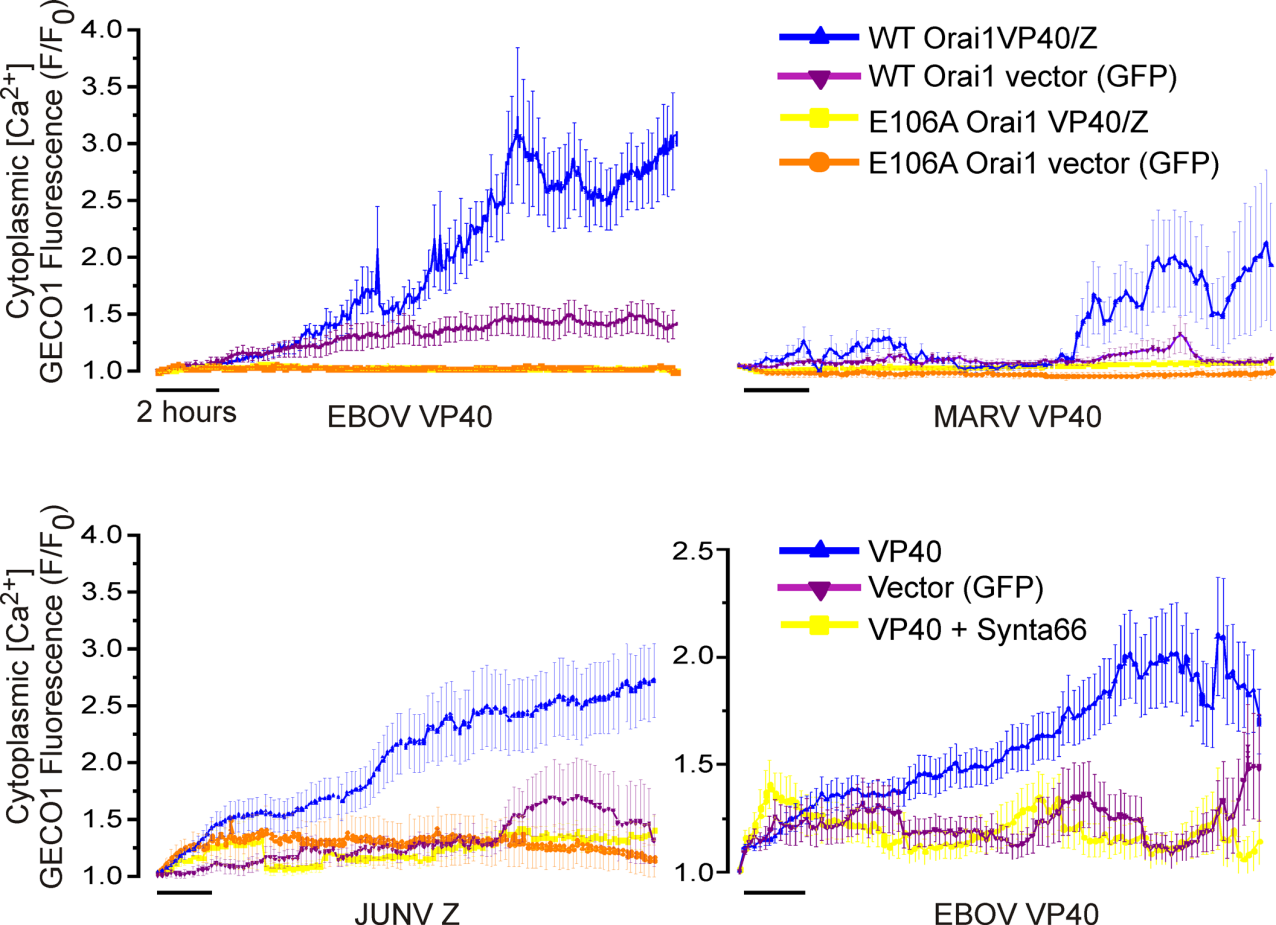
Viral matrix protein expression mobilizes cytoplasmic Ca^2+^. Viral matrix proteins or vector controls and the genetically encoded Ca^2+^ indicator R-GECO-1 were co-expressed in WT HEK293T cells, HEK293T cells stably expressing the dominant negative permeation defective Orai (E106A) mutant, or WT HEK293T cells treated with the Orai inhibitor Synta66 (50 μM, lower right). Cytoplasmic R-GECO-1 fluorescence emission (580nM) was monitored between 6 and 24 hours post transfection from cells cultured in an environmentally controlled chamber on the microscope stage. Each trace represents the average +/- SEM of at least two experiments of at least 20 cells.

While we previously implicated Ca^2+^ in EBOV VP40-dependent VLP generation [32] our initial objective here was to understand if and how hemorrhagic fever virus matrix proteins trigger a change in cytosolic calcium in host cells. To do this we measured intracellular calcium in cells during an extended time course of EBOV and MARV VP40 (eVP40 and mVP40, respectively) and JUNV Z matrix protein-mediated VLP production. Calcium levels (R-GECO-1 fluorescence, [33]) measured in HEK293T cells under physiological conditions for 18–24 hours revealed that eVP40, mVP40, and JUNV Z protein expression each induced a time-dependent increase in Ca^2+^ concentration (Fig 1, blue), while the GFP-vector backbone induced a negligible Ca^2+^ increase that plateaued at a low amplitude or declined to baseline levels (Fig 1, magenta). To probe the role of Orai1 in these responses we performed identical measurements in an HEK293T line that stably expresses a dominant negative mutant Orai1 having a glutamic acid (E) to alanine (A) substitution in its ion selectivity filter (E106A). Incorporation of even a single Orai1 E106A subunit into endogenous WT Orai channels exerts a dominant negative block of its Ca^2+^ permeation [34]. Importantly, both WT and E106A Orai HEK293T cells exhibited a similar transient Ca^2+^ elevation following treatment with the membrane permeant SERCA pump inhibitor thapsigargin in Ca^2+^ free bath solution, indicating that ER stores were intact in E106A Orai1 expressing HEK293T (S1 Fig). The absence of a secondary increase in cytoplasmic Ca^2+^ (S1 Fig, left panel) following reperfusion of HEK293T Orai1 E106A cells with Ca^2+^-containing Ringers solution (S1 Fig, right panel) verified the Orai permeation defect of this line. Significantly, in permeation defective Orai1 E106A cells neither eVP40, mVP40, JUNV Z protein (Fig 1, yellow), nor GFP vector (Fig 1, orange) triggered any change in cytoplasmic Ca^2+^ levels indicating that Ca^2+^ elevations initiated by expression of EBOV, MARV, and JUNV matrix proteins required and resulted from Ca^2+^ entry though Orai channels. Consistent with these results from Orai E106A HEK293T cells and specifically the role of Orai, the Orai inhibitor Synta66 similarly blocked the eVP40-mediated increase in cytoplasmic Ca^2+^ (Fig 1, lower right panel) in WT HEK293T cells.

### Orai1 regulates filovirus and arenavirus VLP production

**Fig 2:**
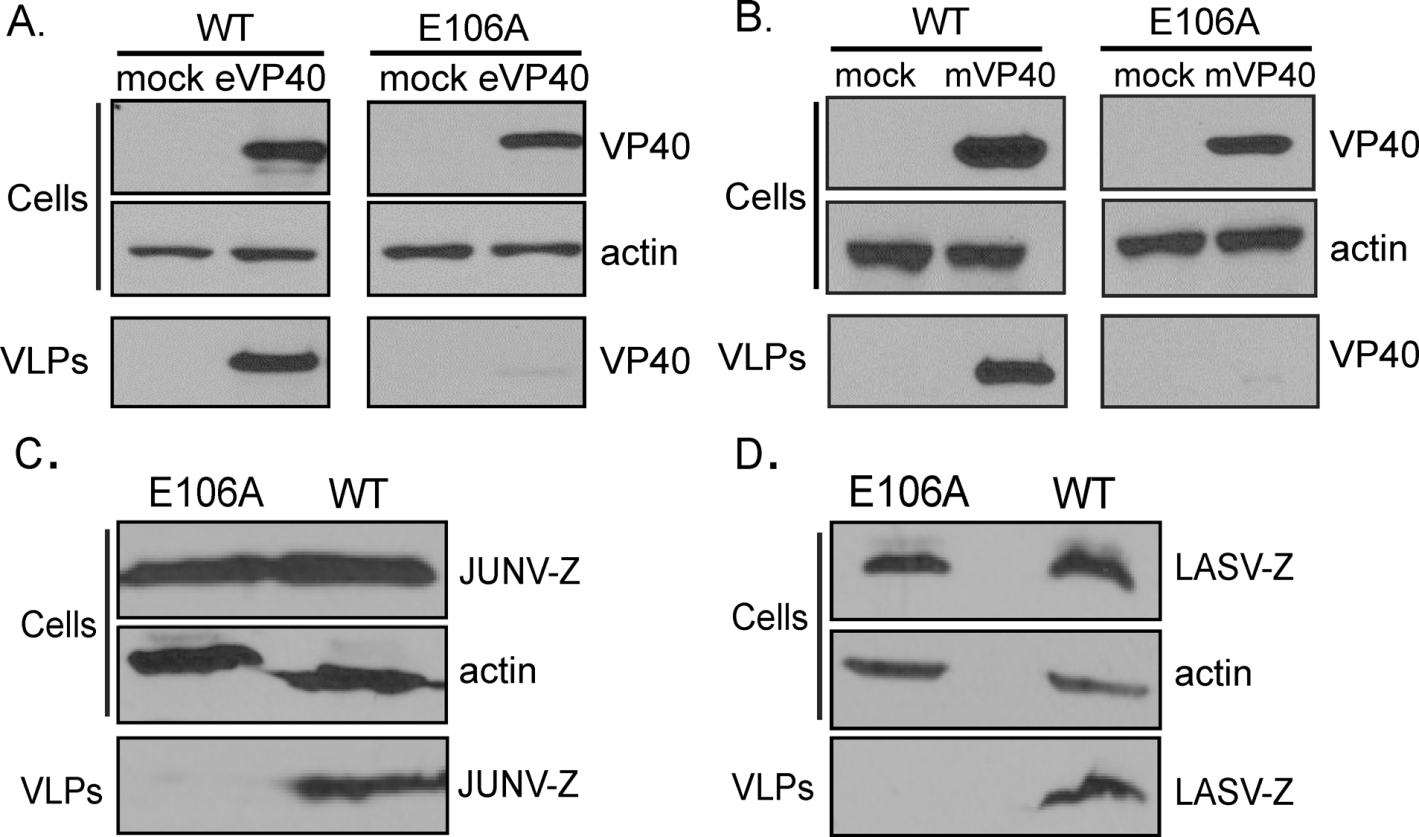
Budding of filovirus and arenavirus VLPs from WT and E106A cells. WT or Orai1 E106A mutant HEK293T cells were transfected as indicated with eVP40 **(A)**, mVP40 **(B)**, JUNV-Z **(C)**, or LASV-Z **(D)** for 24 hours and VP40 or Z protein levels in cells and VLPs were quantified by immunoblot analysis. Expression of cellular actin served as a loading control. Results are representative of three independent experiments.

Given the ability of EBOV, MARV, and JUNV matrix proteins to initiate an Orai-dependent Ca^2+^ signal in HEK293T cells, we assessed the role of Orai1-mediated calcium signals in eVP40, mVP40, LASV Z or JUNV Z mediated VLP production in WT and Orai1 E106A HEK293T cells. Consistent with a role for Ca^2+^ entry via Orai1 in VLP production, we found that Orai1 E106A cells did not support efficient filovirus or arenavirus VLP production (Fig 2). Indeed, levels of eVP40 VLPs from Orai1 E106A cells were ~50 fold lower than that from WT cells (Fig 2A, VLPs). Similarly, production of mVP40 VLPs exhibited an even greater dependence on Orai1-mediated calcium entry (Fig 2B, VLPs), as mVP40 VLPs from Orai1 E106A HEK293T cells were ~100 fold lower than that from WT cells (Fig 2B). Orai similarly regulated JUNV Z (Fig 2C) and LASV Z (Fig 2D) VLP production as both JUNV Z and LASV Z protein-mediated VLP production from E106A cells was ~100 fold lower than that from WT HEK293T cells. In all instances, cellular levels of VP40 or Z were similar in WT and E106A cells, indicating no general requirement for Orai1-mediated Ca^2+^ entry in viral protein expression (Fig 2A–2D; Cells). Together, these data point to a conserved and selective role for Orai-mediated Ca^2+^ entry in hemorrhagic fever virus budding.

### STIM1 expression is required for eVP40 VLP egress

**Fig 3:**
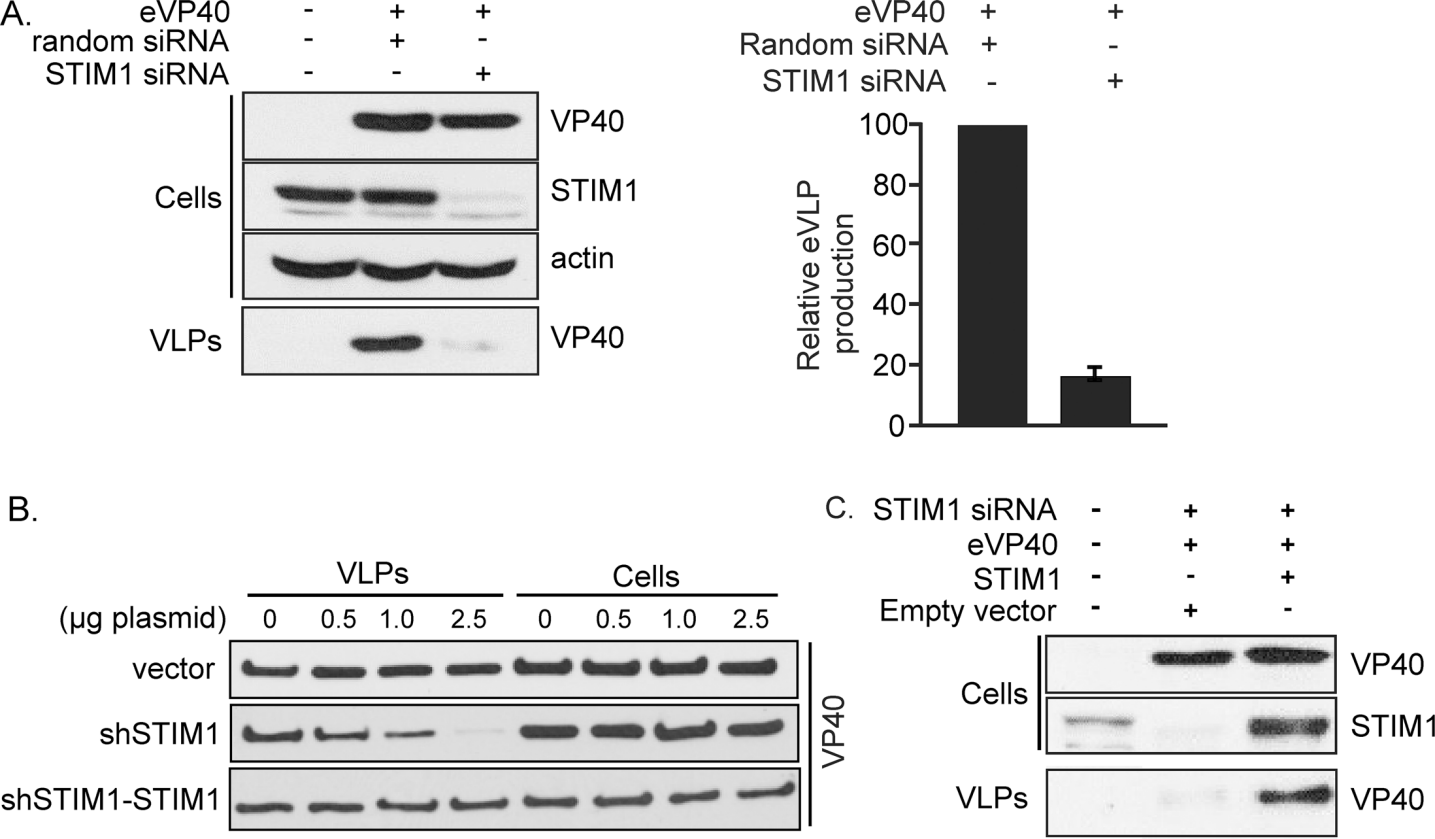
STIM1 suppression inhibits egress of eVP40 VLPs. **A**. VP40 was expressed in control and STIM1 suppressed HEK293T cells as indicated and eVP40 VLP production quantified by immunoblot analysis demonstrates significant inhibition of eVP40 production in STIM1 suppressed cells (n = 4 independent experiments). **B**. Immunoblot analysis of cell extracts and VLPs from HEK293T cells expressing eVP40 in cells co-transfected with indicated amounts of control vector (top panel), STIM1 suppression plasmid (middle panel), or shSTIM1-STIM1 suppression-rescue plasmid (bottom panel). **C**. Cellular VP40 and STIM1 levels were measured in mock-transfected HEK293T cells, or cells transfected with the indicated plasmids and siRNAs. Results are representative of three independent experiments.

Implicit in this common critical role for Orai1-mediated Ca^2+^ entry in EBOV, MARV, JUNV, and LASV VLP production is an upstream requirement for STIM1, the only known trigger for Orai activation in mammalian cells. STIM1 is a single pass ER membrane protein whose activity is regulated by ER Ca^2+^ binding. Ca^2+^ dissociation from STIM1 following a decrease in ER concentration triggers a N-terminal conformational change that initiates its multimerization and relocalization within the ER membrane to domains juxtaposed to the plasma membrane [35–37]. The resulting subplasmalemmal STIM1 clusters physically activate Orai channels to allow extracellular Ca^2+^ to enter the cell [25]. Using eVP40 VLP budding as our model, we probed the role of STIM1 in VLP formation by assessing VLP production from STIM1 suppressed HEK293T cells. eVP40 VLP budding from STIM1 suppressed cells was reduced by approximately 10 fold relative to that from cells receiving random siRNAs or no siRNA (Fig 3A), and the loss of STIM1 had no effect on cellular expression of eVP40 or actin (Fig 3A; Cells). To further confirm this requirement for STIM1 in VLP formation, we utilized a bicistronic vector to suppress endogenous STIM1 (by targeting the 5’ UTR) and rescued its expression with exogenous human STIM1 translated from a shRNA resistant cDNA (shSTIM1-STIM1 plasmid) (Fig 3B). HEK293T WT cells expressing a fixed amount of eVP40 were transfected with increasing amounts of the shSTIM1-suppression vector or empty vector (Fig 3B). While cellular eVP40 expression levels were equivalent under all conditions (Fig 3B, Cells), progressive suppression of STIM1 expression led to a dose-dependent decrease in eVP40 VLP production (Fig 3B, VLPs, middle panel). Importantly, STIM1 re-expression in suppressed cells fully rescued eVP40 VLP production across all levels of STIM1 suppression (Fig 3B, VLPs, bottom panel). Similar to results with STIM1 shRNA, budding of eVP40 VLPs was significantly reduced (Fig 3C, VLP) following siRNA mediated STIM1 suppression (>90%, Fig 3C, middle panel); and over-expression of exogenous STIM1 restored eVP40 VLP production (Fig 3C). Taken together with results from experiments performed on E106A HEK293T cells, these data definitively establish a role for STIM1/Orai dependent Ca^2+^ signals in regulation of VLP egress.

### Pharmacological block of Orai channels inhibits eVP40 and mVP40 VLP production

**Fig 4:**
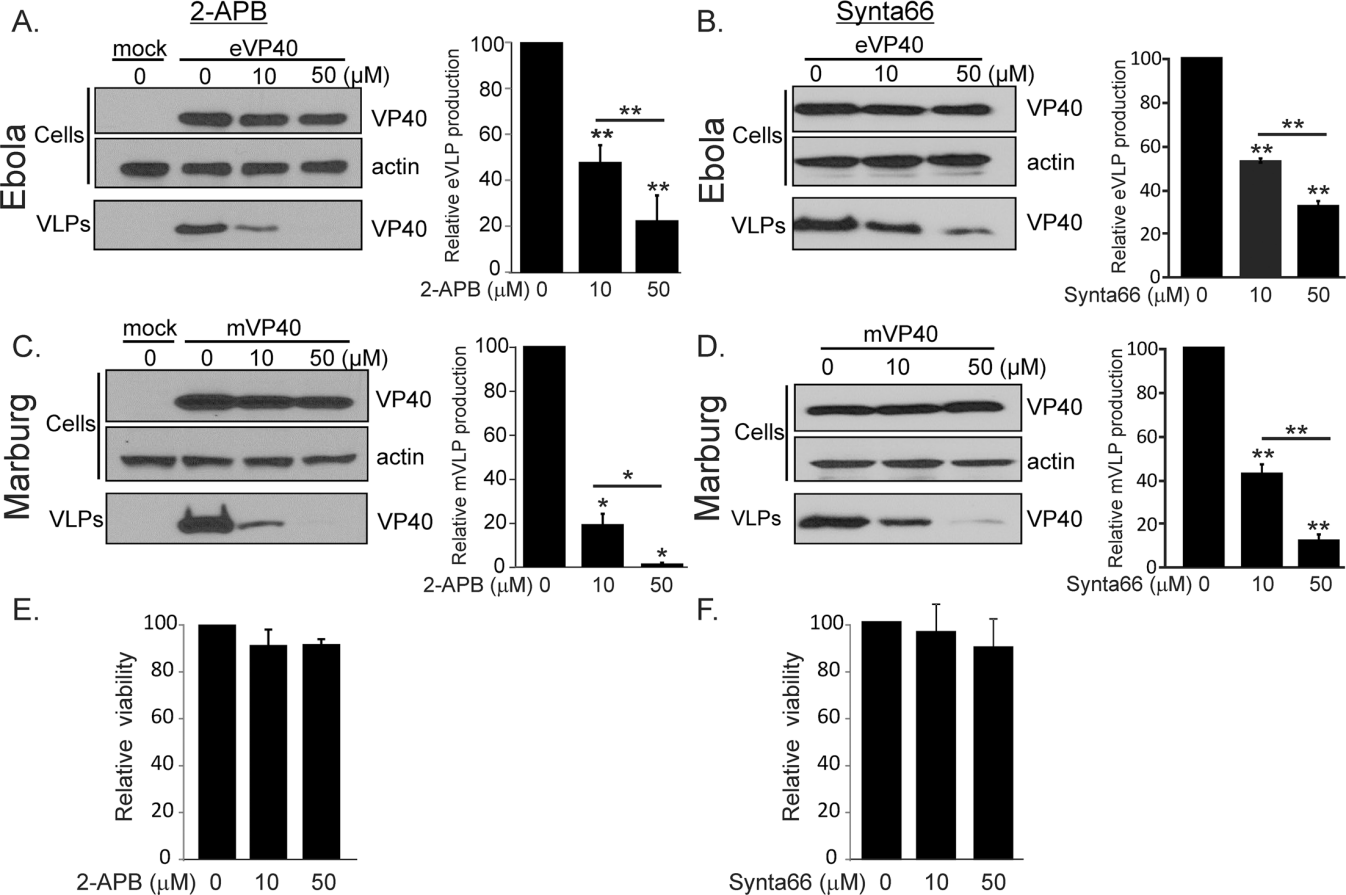
Pharmacological effect of Synta66 and 2-APB on egress of filovirus VLPs. HEK293T expressing eVP40 (**A** and **B**) or mVP40 (**C** and **D**) were treated with indicated concentrations of 2-APB or Synta66. eVP40 and mVP40 levels in cell extracts and VLPs were detected by immunoblot analysis and quantified in VLPs (bar graphs). 2-APB **(E)** and Synta66 **(F)** cytotoxicity was assessed with an MTT viability assay under conditions that mimicked those used for the VLP experiments. In all experiments, cellular actin served as a loading control. Relative protein bands densities were normalized to untreated sample densities (0 μM). Plotted values represent the average (+/- S.E.M) of 3–6 independent experiments and were analyzed with either Welch's t-test or Wilcoxon rank-sum test (*p = 0.01, **p = 0.05).

Genetic approaches outlined above to modulate STIM1 expression and Orai1 permeation establish a novel and common critical role for STIM1 and Orai1 in filovirus and arenavirus budding. Given the broad utility of targeting ion channels to regulate a range of cell physiological functions, we asked whether pharmacological blockade of Orai might represent an effective strategy for regulating filovirus and arenavirus budding. Although high affinity Orai1 blockers for *in vivo* applications are not available at present, we tested several commercially available inhibitors including Synta66 and 2-APB, both of which inhibit Orai-mediated Ca^2+^ entry in HEK293T cells at micromolar levels (10–50 μM) [38, 39] without impacting calcium release from the ER (S2 Fig). Both 2-APB and Synta66 inhibited eVP40- (Fig 4A and 4B) and mVP40-induced (Fig 4C and 4D) VLP production with identical potency as inhibition of Orai-mediated calcium entry, and neither drug affected cellular expression of VP40 or actin (Fig 4A–4D). A concentration of 2-APB that fully blocks Orai1 channels (50 μM) [38] inhibited eVP40 VLP production (Fig 4A, right panel) by ~5 fold and mVP40 VLP production (Fig 4C, right panel) by ~50 fold. Likewise, Synta66 (50μM) substantially inhibited eVP40 (~5 fold) and mVP40 VLP (~10 fold) production (Fig 4B and 4D) with no effect on steady state levels of cellular VP40 or actin and without altering membrane localization of viral proteins (S3 Fig). Importantly, as neither 2-APB (Fig 4E) nor Synta66 (Fig 4F) exerted cytotoxic effects on cells under conditions of these measurements (cell viability, cellular production of VP40, or VP40 membrane localization, Fig 4 and S3 Fig), their anti-budding activity can be attributed to inhibition of Orai-mediated Ca^2+^ entry. Finally, an additional Orai selective inhibitor (RO2959 [40]), which recently became commercially available, also blocks eVP40 VLP formation (~10-fold) with a potency that parallels its inhibition of calcium permeation of the channel (S4 Fig, IC_50_ = ~2.5μM). Thus, the sensitivity of budding to three chemically distinct Orai inhibitors, at the same concentration that blocks calcium permeation of Orai, further substantiates the critical role of Orai-mediated Ca^2+^ entry in VLP production.

### Pharmacological block of Orai channels inhibits arenavirus budding

**Fig 5:**
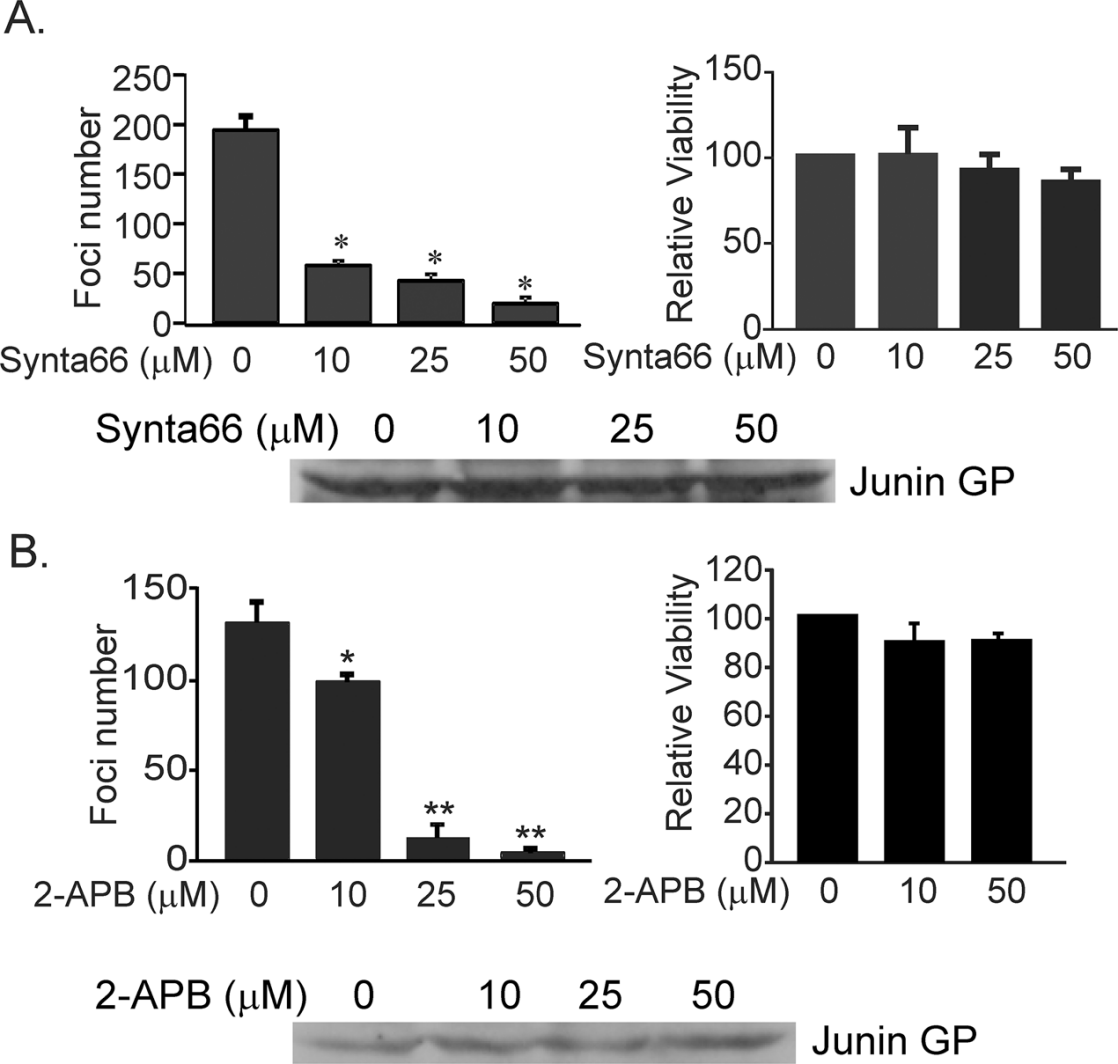
Synta66 and 2-APB inhibit egress of live JUNV (Candid-1 strain) from VeroE6 cells. JUNV foci were visualized and the number of all detectable foci was plotted for each Synta66 **(A)** or 2-APB concentration **(B)**. Statistical significance was analyzed by one way ANOVA (*p < 0.01 for Synta66) (*p < 0.05, **p < 0.01 for 2-APB). MTT based cell viability measurements demonstrate no toxicity of Synta66 **(A)** or 2-APB **(B)** under identical conditions as those used for JUNV infection of VeroE6 cells. Each value is normalized to cells treated with DMSO alone. Comparable levels of JUNV GP were detected by immunoblot analysis in JUNV infected VeroE6 cells in the absence (DMSO alone) or presence of Synta66 **(A)** or 2-APB **(B)**.

We next sought to validate VLP findings by examining the effect of the Orai1 inhibitors Synta66 and 2-APB on budding of the live attenuated Candid-1 JUNV vaccine strain [41, 42]. Briefly, VeroE6 cells infected with live attenuated Candid-1 JUNV were cultured in the absence or presence of Orai inhibitors, and infectious virions produced from these cells were quantified in a focus forming assay (Fig 5). Enumeration of JUNV foci revealed a statistically significant, dose-dependent reduction in JUNV virus production following treatment with Synta66 (Fig 5A) or 2-APB (Fig 5B). Moreover, neither compound affected the viability of cells cultured under conditions mimicking those used for JUNV infection experiments (Fig 5A and 5B, right panels), nor affected the synthesis of JUNV GP in infected VeroE6 cells at any concentration tested (Fig 5A and 5B, Western blots). Together, these findings corroborate results of VLP budding assays (Fig 2) and demonstrate that Orai1-mediated calcium entry is required for efficient budding of infectious JUNV.

### Orai1 regulates budding of infectious pathogenic strains of EBOV, MARV, LASV, and JUNV

**Fig 6:**
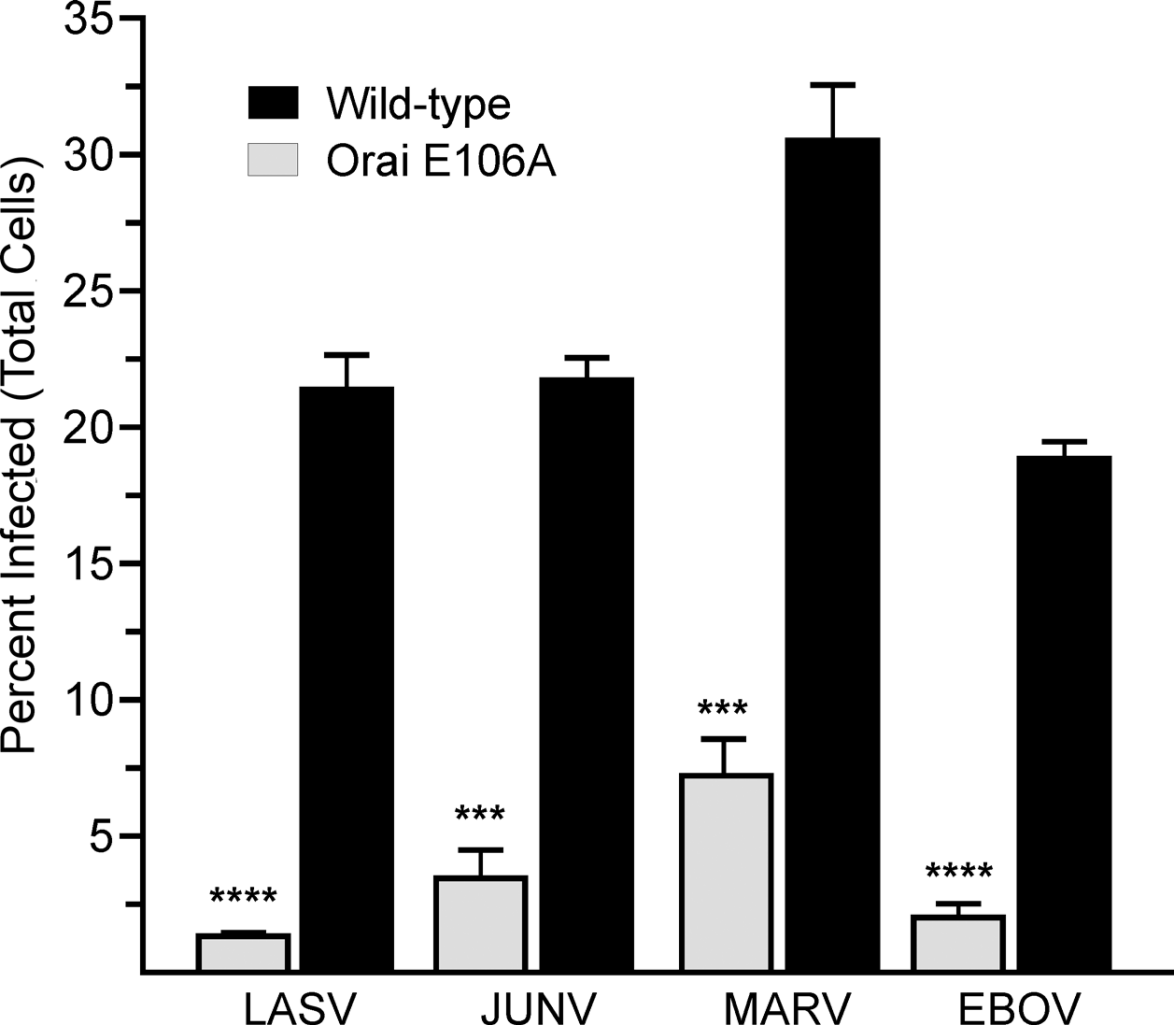
Genetic inactivation of Orai Ca^2+^ permeation inhibits egress of authentic filoviruses and arenaviruses. Wild type HEK293T cells and a Ca^2+^ permeation defective HEK293T line that stably overexpresses the dominant negative Orai E106A mutant were infected with LASV (MOI 0.05), JUNV (MOI 0.1), MARV (MOI 0.1), or EBOV (MOI 0.5). Cellular virus levels were detected by immunofluorescence staining of fixed cells at 72 (LASV) or 96 (JUNV, MARV, EBOV) hours post infection. The percent of infected cells relative to total viable cells per condition is plotted and error bars represent standard error of mean (SEM). Statistical significance was determined by student t test, two-tailed (*** p < 0.0001, **** p < 0.0001 as indicated)

Based on the general requirement we identify for Orai channels in filovirus and arenavirus VLP production and JUNV (Candid-1) budding, we next sought to determine whether Orai channels regulate spread of infectious pathogenic strains of EBOV, MARV, LASV, and JUNV. We first examined viral spread, an indicator of efficient viral budding, in HEK293T cells that constitutively express the dominant negative permeation defective variant of Orai1 (E106A) used in VLP assays described above (Fig 2). These cells were infected at a low multiplicity of infection (MOI), which resulted in the infection of approximately 2–5% of the cells. Cells were then incubated for a period of time that equates to several rounds of viral replication, allowing us to assess viral spread. We observed that the percent of Orai1 E106A expressing cells infected with live BSL-4 variants of EBOV, MARV, JUNV, or LASV was significantly lower than Orai WT cells infected with the same viruses (Fig 6). These results are consistent with a role for Orai in the spread of infectious filoviruses and arenaviruses.

We next assessed the effect of the Orai blocker Synta66 on the spread of these BSL-4 pathogens, because it is a more consistent Orai blocker than 2-APB. Viral spread was assessed by infecting HeLa cells with LASV, JUNV, MARV, or EBOV at a low MOI and then treating with vehicle or Synta66 at the indicated concentrations beginning 1 hour post infection and for the duration of experiments. Seventy two (LASV, JUNV) or 96 (MARV, EBOV) hours post infection we quantified the percentage of cells infected with virus.

**Fig 7:**
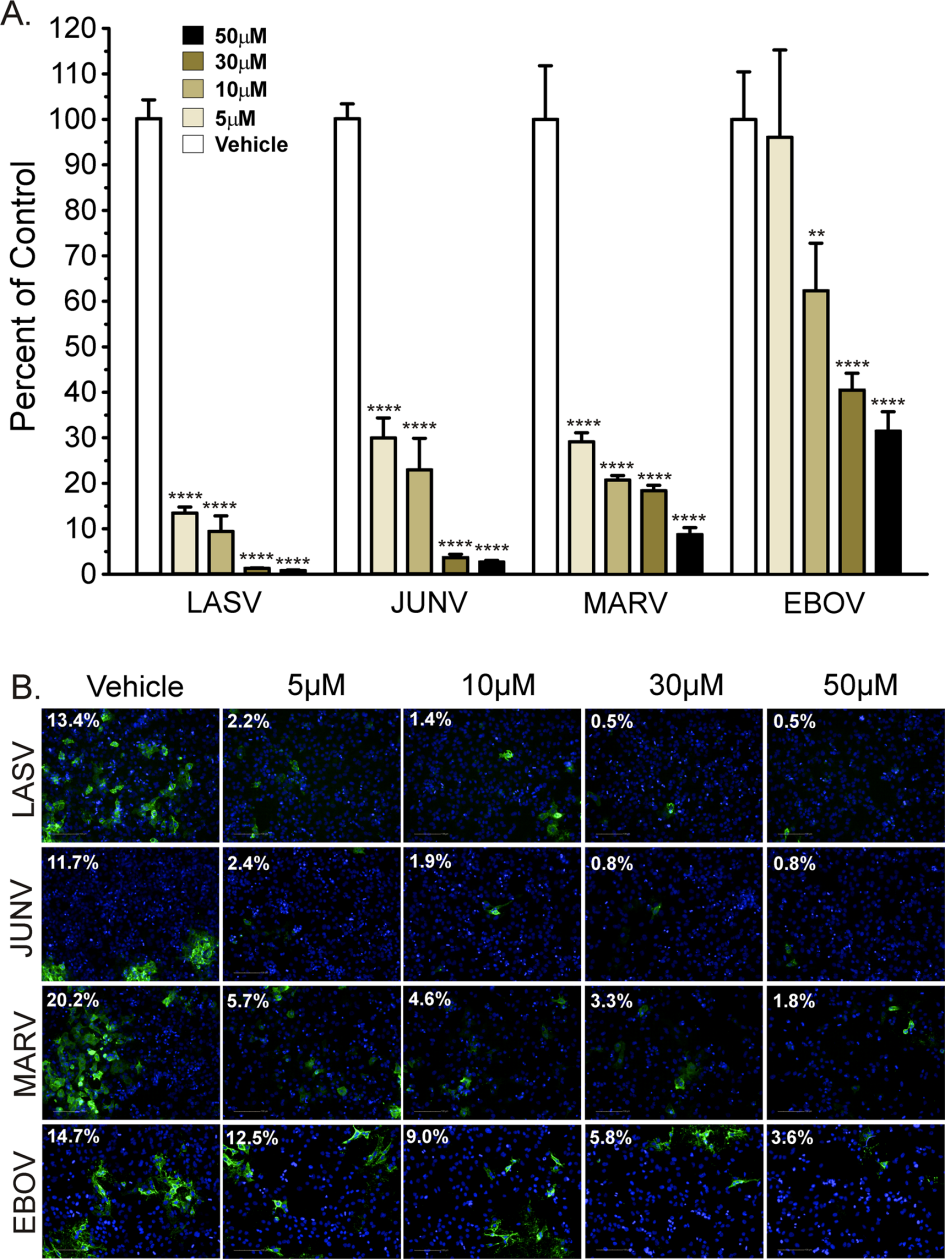
Synta66 inhibits egress of authentic filoviruses and arenaviruses. **A**. HeLa cells were infected with LASV (MOI 0.01), JUNV (MOI 0.1), MARV (MOI 0.1), or EBOV (MOI 0.1) and treated with Synta66 at indicated concentrations. Cellular virus levels were detected by immunofluorescence staining of fixed cells at 72 (LASV, JUNV) or 96 (MARV, EBOV) hours post infection with virus specific antibodies. The percent of cells infected (relative to total cells) was determined using Harmony High Content Imaging and Analysis software (PerkinElmer). Data is expressed relative to vehicle (DMSO) control treated cells for each virus. The percent of infected cells for vehicle control treatment was as follows (LASV = 12% ± 2.69%, JUNV = 9% ± 1.11%, MARV = 20% ± 1.92%, EBOV = 15% ± 1.55%). Error bars indicate standard error of mean (SEM) and statistical significance was determined by two way ANOVA with Bonferroni multiple comparisons (** p < 0.01, **** p < 0.0001). **B**. Representative images from a single live virus experiment demonstrate Synta66 dose (0, 5, 10, 30, 50μM) dependent inhibition of virus spread. For each condition, respective viruses were detected with virus specific antibodies (green). The value in the upper left hand corner of each image is the percentage of total cells infected. For each condition, the total number of cells was determined by staining nuclear DNA with Hoechst DNA dye.

For each virus, we observed a significant Synta66 dose-dependent decrease in the percentage of cells infected (Fig 7A). Consistent with inhibition of viral spread, we also observed a general decrease in the number and size of infected cell clusters with increasing Synta66 concentration (Fig 7B). Similar to the more potent inhibition by Synta66 of mVP40- versus eVP40-mediated VLP production (Fig 4), Synta66 also exerted more potent inhibition of live MARV than EBOV. Interestingly, the spread of both arenaviruses was more sensitive to Orai inhibition than either filovirus (Fig 7A). In general, cultures treated with higher concentrations of Synta66 and for a prolonged period of time (72–96 hours) contained fewer cells than vehicle control treated cultures as measured by the number of nuclei (Fig 7B). For this reason, we normalized the results as the percent of cells infected at the time of analysis for each condition. The decrease in cell numbers, however, does not appear to reflect toxicity (see Figs 4F and 5A). Indeed, as HeLa cells autonomously divide, fewer cells more likely reflects an effect of prolonged Synta66 treatment on cell proliferation. Nonetheless, we evaluated Synta66 induced toxicity by two separate methodologies. Cell-titer Glo “viability” measurements revealed that prolonged Synta66 produced a dose-dependent decrease in ATP (S5 Fig) that is attributed to a decrease in the overall number of cells in cultures and not an effect of Synta66 on cell viability (see Fig 7B). We then utilized an Alamar Blue assay to assess the metabolic health of Synta66 treated cells. Indeed, cellular oxidation-reduction potential of Synta66 treated and vehicle control treated cells were equivalent, confirming comparable metabolic activity (S5 Fig). Thus, while prolonged Synta66 treatment resulted in lower overall cell numbers, those cells that are present are metabolically healthy and are fully capable of producing virus.

**Fig 8:**
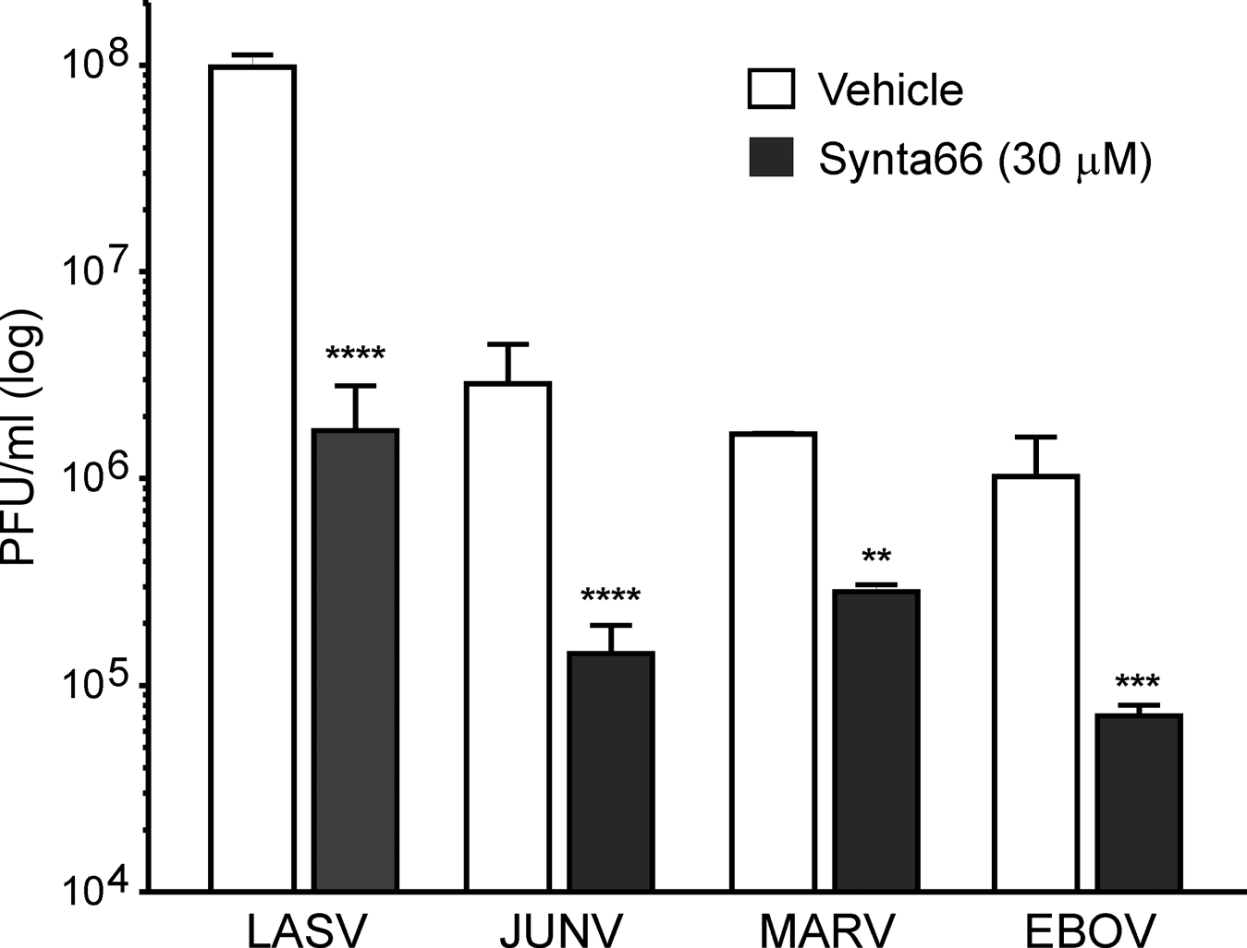
Synta66 decreases the number of viral particles in supernatants. A virus plaque assay used to quantify infectious virions in supernatants of virus infected cells demonstrates that Synta66 (30 μM) significantly inhibits the budding and spread of live LASV, JUNV, MARV, and EBOV. Error bars indicate standard error of mean (SEM) and statistical significance was determined by two way ANOVA with Bonferroni multiple comparisons (**p<0.01, ***p < 0.001, ****p < 0.0001).

We next sought to definitively establish that the effect of Synta66 on virus spread is due to inhibition of virus egress and not entry. We first pretreated HeLa cells with Synta66 and then infected with a high MOI of LASV, JUNV, MARV, or EBOV. Cells were then fixed after only 2–3 viral replication cycles. Infecting with a high MOI and fixing soon after infection ensured that the extent of infection minimally involves spread between cells and rather reflects the extent of primary infection. Under these high MOI conditions, we observed relatively little effect of Synta66 on infection levels with only modest inhibition of infection evident at high Synta66 concentrations (S6 Fig). Further confirmation that Synta66 blocks egress of live filoviruses and arenaviruses was obtained by assessing the amount of virus released into culture supernatants. Supernatants were collected between 48 and 96 hours post-infection with JUNV, LASV, MARV, or EBOV from Synta66 or vehicle treated HeLa cells. Consistent with all of our VLP (Figs 2–4) and live virus data (Figs 5, 6, 7A and 7B) we found that Synta66 (30μM) significantly reduced the titer of infectious Lassa, Junín, Marburg, and Ebola virion particles in culture supernatants (Fig 8). Taken together, this data provide a clear and comprehensive demonstration that Synta66 treatment significantly impairs authentic filovirus and arenavirus budding and release from infected cells.

In summary, our results clearly establish that 1) Orai1-mediated Ca^2+^ entry is a critical virus-triggered host signal that regulates filovirus and arenavirus budding, and 2) STIM1 and Orai1 represent novel targets for broad-spectrum control of these emerging and often fatal viruses. Indeed, the conserved role for Orai mediated calcium entry among these four hemorrhagic fever viruses raises the interesting possibility that Orai inhibitors may have general utility for broad spectrum control of these and other enveloped RNA viruses that bud by similar Ca^2+^ dependent mechanisms.

## Discussion

The recent catastrophic outbreak of EBOV in West Africa highlights the need to develop therapeutics for EBOV and other hemorrhagic fever viruses. Indeed, much progress has been made toward the development of candidate vaccines and therapies against EBOV that are currently in clinical trials. Nevertheless, it is critically important that we improve our understanding of the mechanisms of hemorrhagic fever virus pathogenesis not only to identify novel viral targets, but also to identify host targets and common mechanisms that these viruses require for completion of their life cycles as these could lead to the development of broad spectrum host oriented therapeutics.

A key advantage of therapeutics that target conserved host pathways required broadly by families of viruses for transmission is the potential for broad spectrum efficacy compared with drugs that target strain specific viral targets. Moreover, host targets should be essentially immutable and thereby insensitive to selective pressures that normally allow pathogens to develop drug resistance [3, 12–23]. Here we focused on the second messenger Ca^2+^ and the host proteins responsible for its mobilization and asked whether calcium signals within host cells orchestrate virus assembly and budding.

While calcium has been implicated generally in EBOV and HIV-1 budding [32, 43, 44], previous efforts have not addressed if and how matrix proteins encoded by filoviruses or arenaviruses might trigger changes in Ca^2+^ concentration in host cells. Herein, we demonstrate for the first time that the filovirus matrix protein VP40 and JUNV Z protein trigger STIM1/Orai activation and that the resulting influx of extracellular Ca^2+^ controls both VLP formation and production of infectious filovirus and arenavirus progeny. Moreover, using Orai channel inhibitors, Orai permeation defective lines, and by suppressing STIM1 expression, we establish STIM1 and Orai as effective host targets for pharmacological regulation of virus egress. It should be noted; however, that we cannot rule out a role for other Orai isoforms (Orai2 and Orai3) in to the residual budding observed for live virus from E106A or Synta66 treated cells.

While we have established a critical role for Orai-mediated calcium entry in budding of hemorrhagic fever viruses, the mechanism by which Ca^2+^ does so remains an important question and the focus of ongoing efforts. Indeed, a number of critical steps implicated in efficient budding of enveloped RNA viruses have been linked to cellular Ca^2+^ signals, including the activation and localization of specific ESCRT components. Although not absolutely required, the ESCRT pathway has been shown to play a key role in efficient budding of a plethora of RNA viruses including filoviruses, arenaviruses, rhabdoviruses, and retroviruses [5]. It is tempting to speculate that the observed calcium regulation of budding described here may be linked mechanistically to the role of ESCRT during virus egress. For example, the structure, activation, and interactions of ESCRT-related proteins such as Tsg101, Nedd4, and Alix have been shown to be regulated in part by calcium [44–47]. Additionally, given that Ca^2+^ control of membrane repair reflects ESCRT induced shedding of damaged membrane [48], one might also speculate that Ca^2+^ dependent mechanisms are similarly triggered by insertion of viral proteins in the plasma membrane. Studies underway are thus focused on determining whether Ca^2+^ controls budding through regulation of ESCRT pathway function.

STIM1 and Orai1 mediated Ca^2+^ signals have been implicated in distinct steps of the life cycle of other viruses including the replication of Rotaviruses, which are non-enveloped RNA viruses that do not bud from the plasma membrane. Constitutive STIM1 (and Orai1) activation observed in rotavirus-infected cells reflects an effect of its nonstructural protein 4 (NSP4) on endoplasmic reticulum Ca^2+^ permeability [49]. Indeed, ongoing efforts within our group to understand the mechanisms by which hemorrhagic fever virus matrix proteins trigger STIM1/Orai activation include testing whether VP40 might likewise trigger Ca^2+^ leak from the ER by inhibiting SERCA pump activity. Furthermore, Ca^2+^ influx also seems to regulate entry of West Nile virus [50, 51], Coxsackievirus [52, 53], Hepatitis B virus [54], and Epstein Barr virus [55, 56].

Recently it was shown that subunits of a functionally distinct family of voltage-gated calcium channels (VDCCs) also play a role in JUNV and Mouse Mammary Tumor pseudovirus entry and infection [57] and that the VDCC blockers nifedipine and verapamil suppressed host cell entry by these viruses. Surprisingly; however, in this instance the involvement of VDCC subunits seemed to be distinct from any role in regulating Ca^2+^ levels. How VDCC inhibitors might operate independently of any action on VDCC Ca^2+^ permeation is unclear, but could reflect the promiscuous affinity of VDCC inhibitors for channels including voltage-gated potassium (Kv) channels. Indeed, verapamil and nifedipine also block voltage-gated potassium channels that set the membrane potential of non-excitable cells [58, 59]. Depolarization of the plasma membrane as a result of Kv channel blockade could indirectly block calcium entry by dissipating the electrical driving force (membrane potential) required for calcium permeation of Orai [60–62].

While these studies cumulatively point to additional roles for Orai1-mediated and independent Ca^2+^ influx in steps of infection and replication used by a range of disparate viruses, these roles are distinct from the selective requirement we identify for Orai-dependent calcium entry in budding of filoviruses and arenaviruses. However, Orai might represent a conserved target for regulating budding of additional enveloped RNA viruses, including retroviruses such as HIV-1, which buds by similar mechanisms. Indeed, similar to hemorrhagic fever viruses, the HIV-1 matrix protein Gag directs HIV-1 budding in part, via well-characterized L-domain interactions with ESCRT proteins, and Gag mediated VLP formation also exhibits dependence on Ca^2+^ regulation [43]. Further study is needed to fully assess the role for calcium in the HIV-1 lifecycle because, unlike filoviruses and arenaviruses, Gag-mediated VLP production was found to be insensitive to high concentrations of 2-APB (up to 200uM) that fully block Ca^2+^ permeation of Orai channels [44].

In conclusion, we provide the first direct evidence that host Ca^2+^ signals, triggered by virus activation of STIM1 and Orai, are among key host mechanisms that orchestrate late steps of filovirus and arenavirus assembly and budding. Importantly, from a therapeutic perspective, Orai channels are ubiquitously expressed and like ion channels in general, they represent pharmacologically accessible (cell surface) therapeutic targets. While Orai1 inhibitors by themselves appear to have broad spectrum efficacy, an exciting possibility raised by our results is that drug cocktails formulated to target sequential steps in the virus life cycle, including entry, L-domain/host interactions, and other steps involved in budding, could produce enhanced potency, coverage and efficacy over approaches targeting any one host dependent step in the virus life cycle. Thus, while other calcium channel modulators identified may have distinct targets and even calcium independent effects, they may synergize with Orai1, and also L-domain inhibitors we’ve described previously that block VP40 and Z protein L-domain interactions with host Nedd4 and Tsg101 [42, 63].

Finally, the ability of certain individuals to survive hemorrhagic fever virus infection seems to reflect their capacity to mount a robust anti-viral immune response. In the context of the severity and the acute nature of these viral diseases, the impact of side effects and even minor effects on cell proliferation that might be associated with long term administration of Orai inhibitors that would be required for immune suppression and immune modulation, may be tolerable in the context of infection with these highly pathogenic and often fatal viruses. Indeed, there is no evidence from murine models that the loss of STIM or Orai activity or function would affect antigen induced lymphocyte activation required for an antiviral immune response [64, 65]. Thus our prediction is that administration of Orai1 or STIM1 inhibitors, or cocktails that could also include L-domain inhibitors, would slow or dampen virus transmission within and between individuals, and thereby could provide infected individuals additional time needed to mount a protective adaptive immune response. Although Synta66 and the more potent compound RO2959 are no longer being developed as therapeutics, several smaller pharmaceutical companies and academic groups persist in efforts to develop potent Orai1 inhibitors to suppress the pathogenesis of chronic immune-mediated and inflammatory diseases. If and when these become available, direct inhibition of enveloped RNA virus budding from host cells and transmission between individuals may represent an entirely novel use for these channel blockers.

## Materials and Methods

### Cell lines, plasmids, and reagents

HEK293T, HeLa, and VeroE6 cells were maintained in Dulbecco’s modified Eagle’s medium (DMEM) supplemented with 10% fetal calf serum (FCS), penicillin (100 U/ml)/streptomycin (100μg/ml) at 37°C in a humidified 5% CO_2_ incubator. The stable HEK293T Orai1 E106A mutant-expressing cell line was maintained in DMEM with 10% FCS, penicillin (100 U/ml)/streptomycin (100μg/ml) in the presence of 500 μg/ml of G418. HeLa cells were maintained in Minimal Essential Medium (MEM) with 5% fetal bovine serum, penicillin (100 U/ml)/streptomycin (100μg/ml) at 37°C in a humidified 5% CO_2_ incubator. The pCAGGS based plasmids expressing EBOV VP40, MARV VP40, JUNV Z, LASV Z, and GFP-eVP40 have been described previously [42, 63, 66]. mVP40 and JUNZ Z protein are flag tagged while eVP40 was detected using an anti-eVP40 polyclonal antibody previously described [67]. mVP40 and JUNV Z protein were detected with an anti-flag monoclonal antibody (Sigma-Aldrich), and STIM1 was detected with a rabbit anti-STIM1 specific polyclonal antibody (gift of Dan Billadeau, Mayo Clinic). 2-aminoethoxy diphenyl borate (2-APB, Sigma Aldrich), Synta66, and RO2959 (Glixx Labs, Southborough, MA) were freshly prepared from stock solutions in DMSO.

### Viability assays

Cell viability in VLP budding and live virus infection assays was examined using an MTT assay (Amresco). 5×10^3^ HEK293T or VeroE6 cells were plated in collagen-coated 96-well tissue culture plates in triplicate. Cells were transfected with empty vector using Lipofectamine for 6 hours, and incubated in serum-free or 2% FCS in phenol-red-free OPTI-MEM in the presence of Synta66 or 2-APB at the indicated concentrations for 20 hours, which mimics the transfection and treatment conditions for VP40 VLP budding. 20μl of MTT solution (5mg/ml in PBS) was added into each well and cells were incubated for 3.5 hours. Media was discarded and 150 μl DMSO was added. Absorbance was determined by spectrophotometry using a wavelength of 590 nm. For experiments with BSL-4 variants of filoviruses and arenaviruses, HeLa cells were seeded in 96 well plates ~24 hours prior to addition of Synta66 or vehicle control at indicated concentrations. Cells were incubated for 72 or 96 hour at 37°C in a humidified 5% CO_2_ incubator and viability was assessed using either CellTiter-Glo assay (Promega), or AlamarBlue assay (Life Technologies), in accordance with manufacturer’s instructions.

### Calcium imaging

HEK 293T Orai1-wild type and Orai1-E106A mutant cells (kind gift from Dr. Jonathan Soboloff, Temple University) were plated at 5x10^5^ cells/well in a 2-chambered Lab-Tek II Chambered #1.5 slide (Nunc, Rochester, NY) and grown overnight at 37°C, 5% CO_2_ in Dulbecco’s modified Eagle’s Medium supplemented with 4.5g/L glucose, L-glutamine, sodium pyruvate (Mediatech, Inc., Manassas, VA), 10% FBS (Gibco), and 1% Penicillin/Streptomycin (Gibco). Cells were transfected with 1μg of R-GECO-1 plasmid (Addgene, Cambridge, MA) using Lipofectamine 2000 reagent (Invitrogen) according to manufacturer’s instructions in phenol-red-free OPTI-MEM. The next day, cells were transfected with GFP-eVP40 fusion plasmid (GFP-eVP40) using Lipofectamine 2000 reagent (Invitrogen) according to manufacturer’s instructions in phenol-red-free OPTI-MEM. Six hours post transfection, fresh phenol-red-free OPTI-MEM was added to the wells, and cells were imaged at 37°C and 5% CO_2_ in a custom environmental chamber for the duration of the imaging on a Leica DMI4000 with Yokagawa CSU-X1 Spinning Disk Microscope with a 20X dry objective. Cellular R-GECO-1 fluorescence was imaged every 4 seconds for 1 minute periods, repeated every 10 minutes over 18 hours with a Hamamatsu 16-bit cooled EMCCD camera. Imaging and data analysis were performed using the Metamorph 7.6 imaging suite. Normalized fluorescence intensity (F/F_0_, where F_0_ is calculated as the average fluorescence intensity for the initial 1 minute interval) was calculated for each region of interest (ROI) in the time-series.

### Virus like particle budding assay

WT HEK293T or HEK293T E106A cells were seeded in collagen-coated six-well plates and transfected with 0.5μg of the indicated expression plasmids using Lipofectamine (Invitrogen) and the protocol of the supplier. At 6 hours post-transfection, cells were incubated in serum-free OPTI-MEM media or 2% FCS DMEM for 20–24 hours. Cells were incubated with vehicle (DMSO) alone, Synta66, or 2-APB at the indicated concentrations. Culture medium was harvested and centrifuged at 2500 rpm for 10 minutes to remove cellular debris, layered over a 20% sucrose cushion in STE buffer and centrifuged at 220,000xG for 2 hours at 4°C. The VP40 VLP-containing pellet was suspended in 50 μl of STE buffer at 4°C overnight. Cells were lysed in RIPA buffer as described above. Viral proteins in VLPs and cell lysates was detected by SDS-PAGE and Western-blot using primary rabbit anti-VP40 antibody for Ebola VP40, mouse anti-flag antibody for Marburg VP40, or mouse anti-HA antibody for LASV and JUNV Z followed by an appropriate HRP-conjugated secondary antibody.

### siRNA and shRNA mediated protein suppression

Human STIM1 siRNAs were purchased from Dharmacon SMARTpools. ON-TARGETplus STIM1 siRNA (Thermo SCIENTIFIC) is a mixture of 4 siRNAs to specifically silence the target gene. HEK293T cells in OPTI-MEM media in collagen-coated six-well plate were transfected twice with either control siRNA or STIM1 siRNA at a final concentration of 200nM using Lipofectamine (Invitrogen) at 2-day intervals. The final transfection included both siRNAs and 0.5μg of Ebola VP40 expression plasmid. VLPs and cell lysates were harvested at 48 hours following the last transfection as described above. VP40 protein levels in VLPs and VP40 and STIM1 levels in cell extracts were analyzed by Western-blot with rabbit anti-VP40 antibody, or rabbit anti-STIM1 antibody, followed by anti-rabbit IgG HRP-conjugated secondary antibody. STIM1 suppression and rescue was accomplished using bicistronic vectors developed in house. STIM1 shRNA generated against the 5’ untranslated region of human STIM1 was expressed using the H1 promoter and human STIM1 cDNA expressed from a CMV promoter in the same construct. VLP samples and cell lysates were harvested at 48 hours after last transfection and analyzed by Western-blot.

### Candid-1 Junin virus and infection

The Candid-1 vaccine strain of JUNV was kindly provided by Robert B. Tesh (U.T.M.B., Galveston, TX) via Susan R. Ross (Univ. of Penn., Philadelphia, PA), and was propagated in VeroE6 cells as described previously [41]. For JUNV infection, VeroE6 cells were infected with JUNV (Candid-1) at an MOI of 0.02 for 42 hours at 37°C. Supernatants were removed and the cells were washed 3X with 1X PBS. Cells were then treated with DMSO alone, or the indicated concentrations of Synta66 for an additional 30 hours. Virions were harvested from the supernatant samples as described above for VLPs, and then used to infect fresh monolayers of VeroE6 cells for 48 hours for quantification of all foci detected in 6-well plates using fluorescence microscopy as described previously [42].

### Authentic virus infection of HEK293T cells

For all experiments using authentic viruses, Ebola virus (Kikwit isolate), Marburg virus (Ci67 isolate), Lassa virus (Josiah isolate), and Junin virus (Espindola isolate) were used. Wild-type HEK293T cells and Orai1 E106A mutant-expressing HEK293T cells, seeded in 96 well black plates (Corning BioCoat Cellware, Collagen Type I), were incubated with EBOV (MOI = 0.5), MARV (MOI = 0.1), JUNV (MOI = 0.1), or LASV (MOI = 0.05) in a Biosafety Level 4 laboratory located at USAMRIID. Following 1 hour absorption, virus inoculum was removed and growth media was added. Cells were then incubated at 37°C, 5% CO2, 80% humidity for 72 (LASV) or 96 (EBOV, MARV, JUNV) hours, at which time the cells were washed once with PBS and submerged in 10% formalin prior to removal from the BSL4 laboratory. Formalin was removed and cells were washed 3 times with PBS. For LASV infection only, cells were treated with 300mM NaOH for 20 minutes at room temperature prior to the blocking step. Cells were blocked by adding 3% BSA/PBS to each well and incubating at 37°C for 2 hours. EBOV GP-specific mAb KZ52 (kind gift from Kartik Chandran, Albert Einstein College of Medicine, Bronx, NY), MARV GP-specific mAb 9G4 (USAMRIID), LASV GP-specific mAb 52-161-6 (USAMRIID), and JUNV GP-specific mAb GD01-AG02 (BEI Resources), diluted in 3% BSA/PBS, were added to appropriate wells containing infected cells and incubated at room temperature for 2 hours. Cells were washed 3 times with PBS prior to addition of goat anti-mouse or goat anti-human IgG-AlexaFluor 594 (Invitrogen, Molecular Probes) secondary antibody. Following 1 hour incubation with secondary antibody, cells were washed 3 times prior to addition of Hoechst 33342 (Invitrogen, Molecular Probes) diluted in PBS. Cells were imaged and percent of virus infected cells calculated using the Operetta High Content Imaging System (PerkinElmer) and Harmony High Content Imaging and Analysis Software (PerkinElmer). Statistical significance between wild-type cells and Orai1 E106A mutant cells was determined using student t test, two-tailed.

### Authentic virus infection of Synta66 treated HeLa cells

For immunofluorescence based assays, HeLa cells, seeded in 96 well black plates (Greiner Bio-One Cellcoat), were incubated with EBOV (MOI 0.1), MARV (MOI 0.1), LASV (MOI 0.01), or JUNV (MOI 0.1) for 1 hour at 37°C, 5% CO_2_, 80% humidity. Virus inoculum was removed and cells were washed once with PBS. Synta66 was diluted in HeLa cell culture media at indicated concentrations and added to cells. An equivalent percentage of DMSO in HeLa media served as the vehicle control. Cells were then incubated at 37°C, 5% CO_2_, 80% humidity for 72 (LASV and JUNV) or 96 (EBOV and MARV) hours at which time, the cells were washed once with PBS and submerged in 10% formalin prior to removal from the BSL4 laboratory. As described above, virus specific antibodies were added to appropriate wells containing infected cells and samples processed as previously described, except that goat anti-mouse or goat anti-human IgG-AlexaFluor 488 (Invitrogen, Molecular Probes) was used as the secondary antibody. Statistical significance was determined by two way ANOVA with Bonferroni multiple comparisons relative to vehicle control treated cells. For viral titer analysis, HeLa cells were seeded in 6 well plates ~24 hours prior to infection with LASV (MOI = 0.01), JUNV (MOI = 0.1), MARV (MOI = 0.1), or EBOV (MOI = 0.1). One hour after infection, cells were treated with Synta66 or vehicle control at indicated concentrations. Culture supernatants were collected at 48 (MARV), 72 (LASV, JUNV) or 96 (EBOV) hours and cell debris removed by centrifugation. Viral titers of clarified supernatants were determined by routine plaque assay as previously described [68–70]. All data is a graphical representation of at least two independent, replicate experiments. Statistical significance of log transformed data was determined by two way ANOVA with Bonferroni multiple comparisons relative to vehicle control treated cells.

## Supporting Information

S1 Fig Inhibition of Orai1 calcium permeation in an HEK293T line that expresses Orai1 dominant negative mutant E106A. Cytoplasmic Ca^2+^ levels were measured in HEK293T cells with the fluorescent ratiometric calcium indicator Fura-2. Cells incubated in Ca^2+^ free Ringers solution were treated with the SERCA inhibitor thapsigargin (1μM) to trigger passive depletion of Ca^2+^ from the ER and in order to activate Orai. The transient cytoplasmic Ca^2+^ elevation observed in Ca^2+^ free Ringers solution reflects this ER Ca^2+^ release. While cells that express WT Orai exhibit a secondary Ca^2+^ increase upon perfusion with 2 mM Ca^2+^ Ringers, the absence of Ca^2+^ influx in cells that express Orai E106A confirms the Ca^2+^ permeation blockade in this cell line.
(TIF)

S2 Fig Inhibition of Orai-mediated calcium entry by 2-APB and Synta66. The calcium indicator Fura-2 was used to measure cytoplasmic Ca^2+^ levels in HEK293T cells. Cells bathed in Ca^2+^ free Ringers solution were treated with **(A)** thapsigargin (1μM) or **(B)** ionomycin (1μM) to deplete Ca^2+^ from the ER and activate Orai. The transient response observed in Ca^2+^ free Ringers reflects ER Ca^2+^ release. Consistent with Orai activation, subsequent reperfusion with Ca^2+^ containing Ringers produced a secondary sustained cytoplasmic Ca^2+^ elevation due to Ca^2+^ entry through activated Orai channels (left panels). Both 2-APB (**A**, right panel) and Synta66 (**B**, right panel) blocked Ca^2+^ entry following ER depletion consistent with a block of Orai.
(TIF)

S3 Fig Orai-independent plasma membrane localization of eVP40. HEK293T cells expressing a budding competent GFP-eVP40 fusion protein were cultured for 24 hours in the absence or presence of Synta66 (10 μM) and then fixed and counterstained with CellMask deep red cytoplasmic stain. 3D reconstruction of Z series images of HEK293T cells expressing GFP tagged eVP40 and counterstained with Cell Mask Red (upper panels) demonstrate equivalent membrane localization of eVP40 in untreated and Synta66 treated cells. Similar membrane localization is also evident in representative single confocal sections (bottom panels) from untreated (left) and Synta66 (right) treated cells. Together, these observations support our functional studies that point to a role for Orai1-mediated Ca^2+^ entry in steps of VLP formation that occur subsequent to VP40 membrane localization. For these images the scale bar = 10μm.
(TIF)

S4 Fig RO2959 inhibition of Ca^2+^ entry and eVP40 VLP formation. Cells were initially bathed in Ca^2+^ free Ringers solution and ER Ca^2+^ was depleted with ionomycin to activate plasma membrane Orai channels. The secondary Ca^2+^ elevation following reperfusion with Ca^2+^ containing Ringers solution evident in untreated cells (left trace) reflects entry through activated Orai channels. This secondary increase was blocked by the Orai inhibitor RO29959 (5 μM) (top panel, right trace). Does dependent inhibition of Orai with RO2959 also produced dose dependent inhibition of eVP40 VLP production without inhibiting cellular VP40 expression (bottom panel)
(TIF)

S5 Fig Viability of Synta66 treated HeLa cells. **A**. Cell viability was assessed in HeLa cells treated with indicated concentrations of Synta66. At 72 and 96 hours post treatment, CellTiter-Glo (Promega) assay was performed to measure cellular ATP as an indicator of cell number. **B**. An Alamar Blue viability assay was performed to assess the metabolic activity of HeLa cells treated with Synta66 for 72 or 96 hours. Data is expressed as the percent of vehicle (DMSO) control treated cells. Error bars indicate standard error of mean (+/- SEM) and statistical significance was determined by two way ANOVA with Bonferroni multiple comparisons (**** p < 0.0001).
(TIF)

S6 Fig Synta66 does not affect LASV, JUNV, MARV, or EBOV entry. HeLa cells, seeded in 96 well plates ~24 hours prior to infection, were pretreated with Synta66 at indicated concentration or vehicle for 1 hour. Cells were infected with LASV (MOI = 0.1), JUNV (MOI = 1), MARV (MOI = 1), or EBOV (MOI = 1). One hour after infection, cells were washed and treated with fresh Synta66 or vehicle at indicated concentrations. Cells were fixed at 24 (JUNV, LASV) or 48 (MARV, EBOV) hours post infection and percent of infected cells was determined by immunofluorescence staining with virus specific antibodies. Data is expressed as the percent of vehicle (DMSO) control treated cells and error bars indicate standard error of mean (+/- SEM).
(TIF)
