# Supplementary figures and images for: Calcium Regulation of Hemorrhagic Fever Virus Budding: Mechanistic Implications for Host-Oriented Therapeutic Intervention
Source: PLoS Pathog. 2015 Oct 29;11(10):e1005220. doi: 10.1371/journal.ppat.1005220 (PMC4634230; doi:10.1371/journal.ppat.1005220)

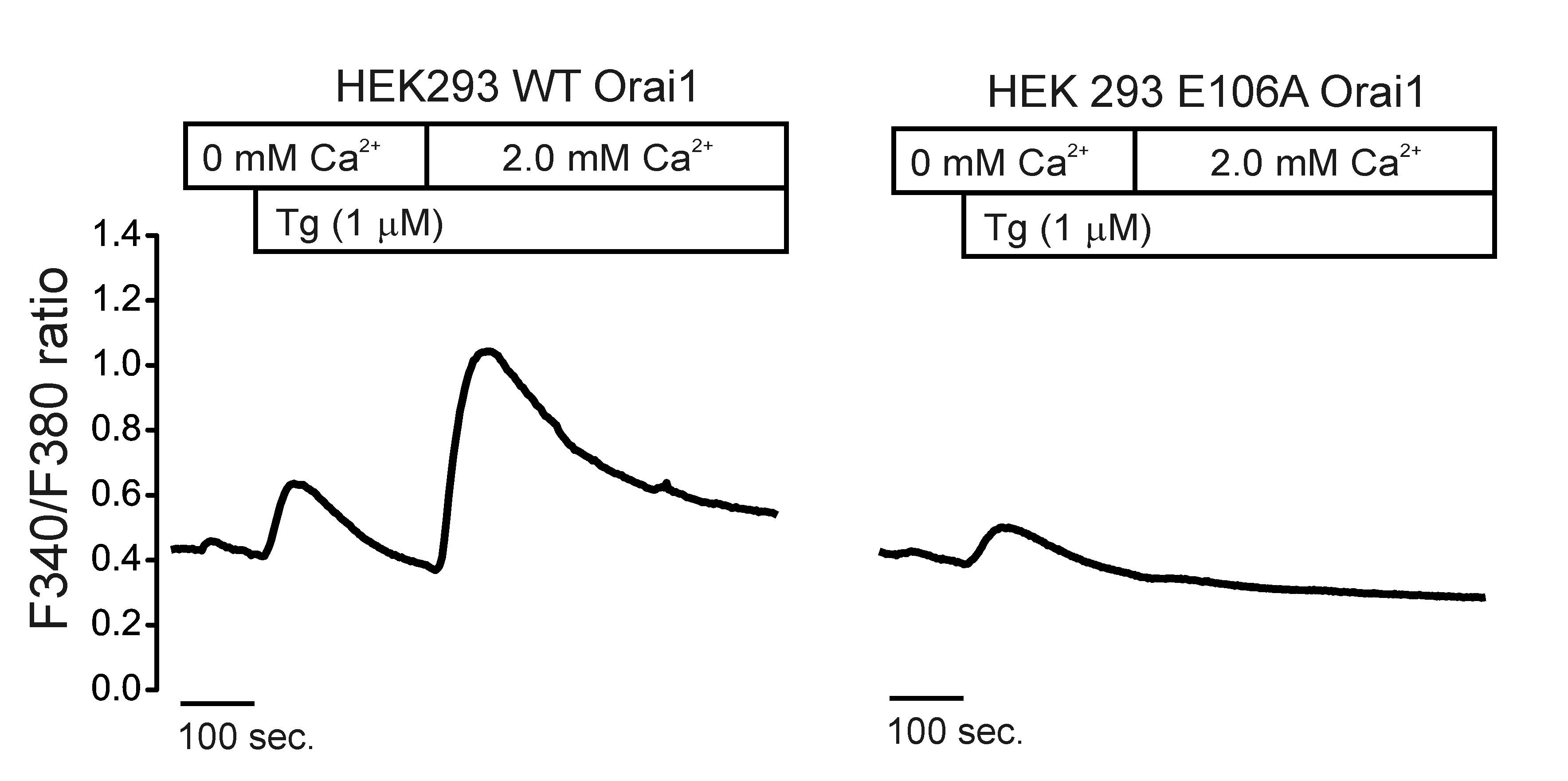

Supplement: S1 Fig — Cytoplasmic Ca2+ levels were measured in HEK293T cells with the fluorescent ratiometric calcium indicator Fura-2. Cells incubated in Ca2+ free Ringers solution were treated with the SERCA inhibitor thapsigargin (1μM) to trigger passive depletion of Ca2+ from the ER and in order to activate Orai. The transient cytoplasmic Ca2+ elevation observed in Ca2+ free Ringers solution reflects this ER Ca2+ release. While cells that express WT Orai exhibit a secondary Ca2+ increase upon perfusion with 2 mM Ca2+ Ringers, the absence of Ca2+ influx in cells that express Orai E106A confirms the Ca2+ permeation blockade in this cell line. (TIF) [file ppat.1005220.s001.TIF]

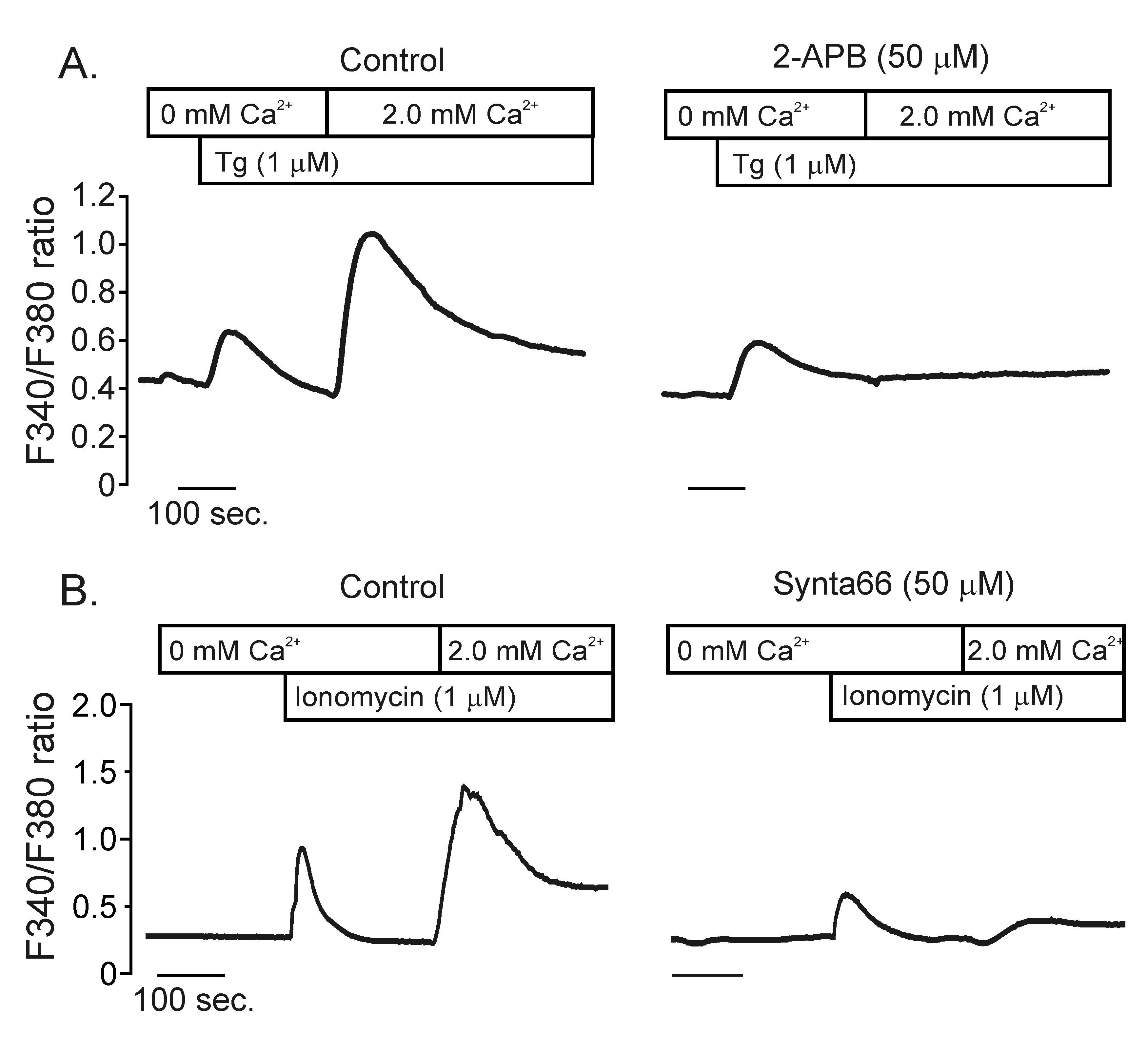

Supplement: S2 Fig — The calcium indicator Fura-2 was used to measure cytoplasmic Ca2+ levels in HEK293T cells. Cells bathed in Ca2+ free Ringers solution were treated with (A) thapsigargin (1μM) or (B) ionomycin (1μM) to deplete Ca2+ from the ER and activate Orai. The transient response observed in Ca2+ free Ringers reflects ER Ca2+ release. Consistent with Orai activation, subsequent reperfusion with Ca2+ containing Ringers produced a secondary sustained cytoplasmic Ca2+ elevation due to Ca2+ entry through activated Orai channels (left panels). Both 2-APB (A, right panel) and Synta66 (B, right panel) blocked Ca2+ entry following ER depletion consistent with a block of Orai. (TIF) [file ppat.1005220.s002.TIF]

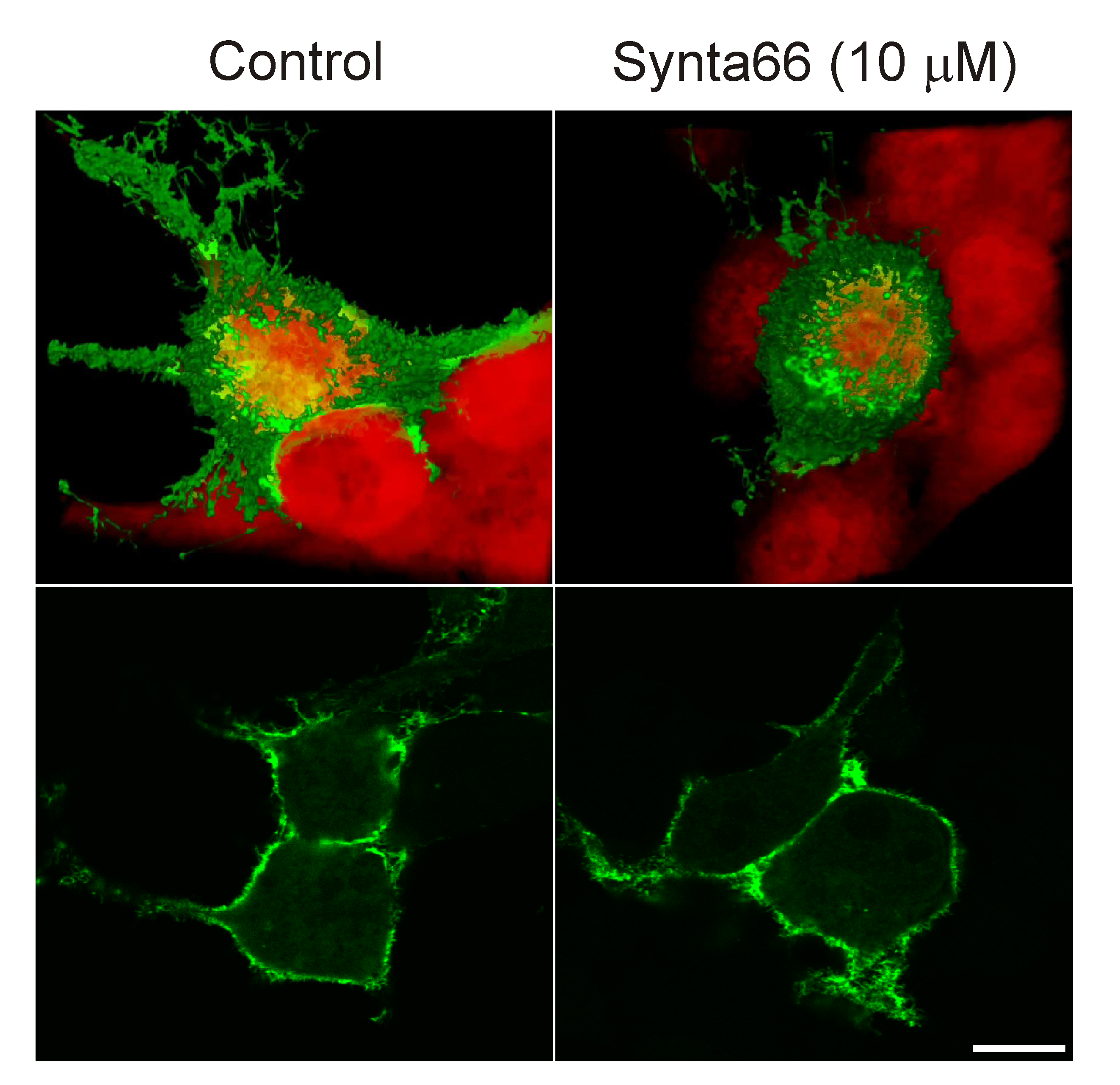

Supplement: S3 Fig — HEK293T cells expressing a budding competent GFP-eVP40 fusion protein were cultured for 24 hours in the absence or presence of Synta66 (10 μM) and then fixed and counterstained with CellMask deep red cytoplasmic stain. 3D reconstruction of Z series images of HEK293T cells expressing GFP tagged eVP40 and counterstained with Cell Mask Red (upper panels) demonstrate equivalent membrane localization of eVP40 in untreated and Synta66 treated cells. Similar membrane localization is also evident in representative single confocal sections (bottom panels) from untreated (left) and Synta66 (right) treated cells. Together, these observations support our functional studies that point to a role for Orai1-mediated Ca2+ entry in steps of VLP formation that occur subsequent to VP40 membrane localization. For these images the scale bar = 10μm. (TIF) [file ppat.1005220.s003.TIF]

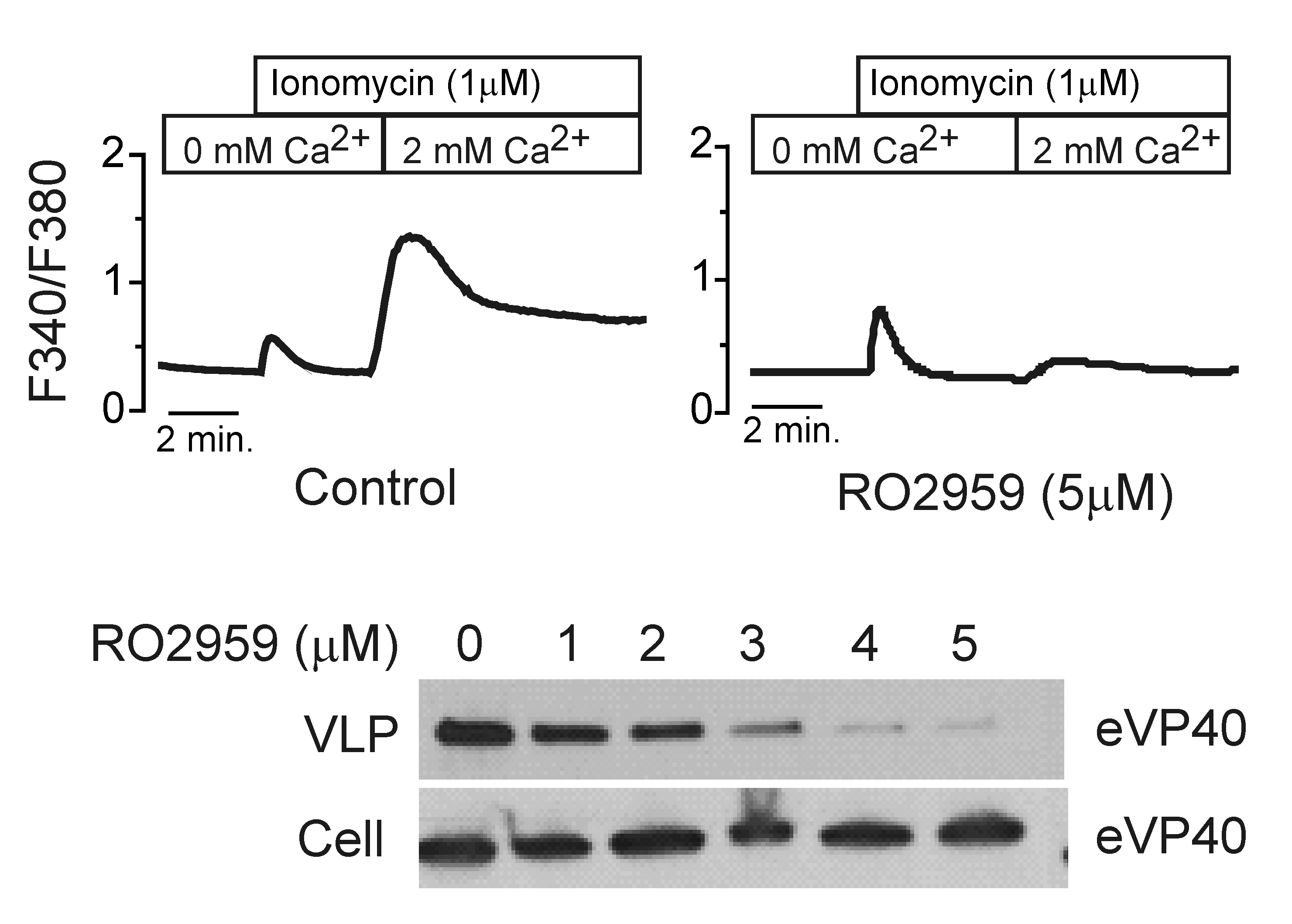

Supplement: S4 Fig — Cells were initially bathed in Ca2+ free Ringers solution and ER Ca2+ was depleted with ionomycin to activate plasma membrane Orai channels. The secondary Ca2+ elevation following reperfusion with Ca2+ containing Ringers solution evident in untreated cells (left trace) reflects entry through activated Orai channels. This secondary increase was blocked by the Orai inhibitor RO29959 (5 μM) (top panel, right trace). Does dependent inhibition of Orai with RO2959 also produced dose dependent inhibition of eVP40 VLP production without inhibiting cellular VP40 expression (bottom panel) (TIF) [file ppat.1005220.s004.TIF]

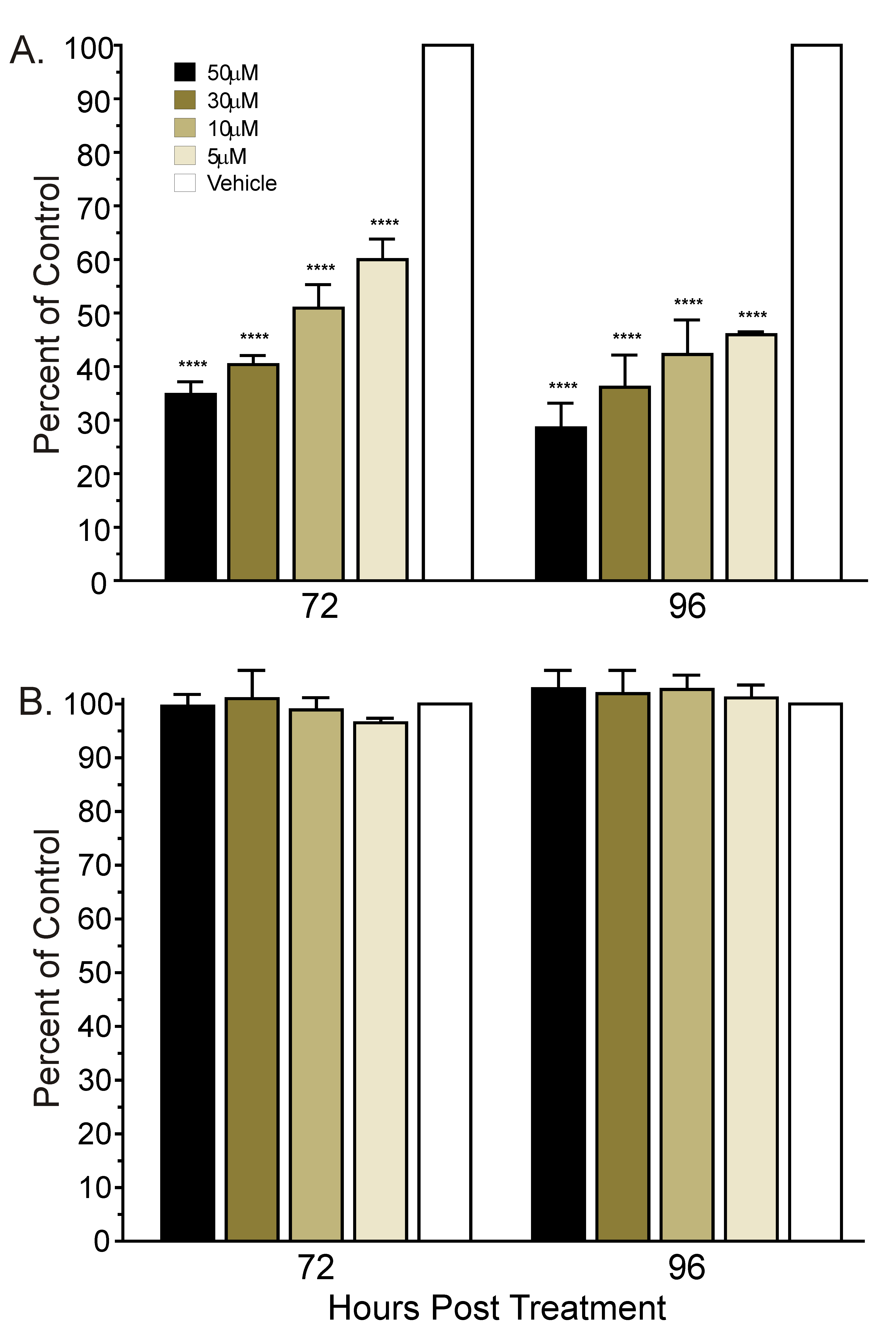

Supplement: S5 Fig — A. Cell viability was assessed in HeLa cells treated with indicated concentrations of Synta66. At 72 and 96 hours post treatment, CellTiter-Glo (Promega) assay was performed to measure cellular ATP as an indicator of cell number. B. An Alamar Blue viability assay was performed to assess the metabolic activity of HeLa cells treated with Synta66 for 72 or 96 hours. Data is expressed as the percent of vehicle (DMSO) control treated cells. Error bars indicate standard error of mean (+/- SEM) and statistical significance was determined by two way ANOVA with Bonferroni multiple comparisons (**** p < 0.0001). (TIF) [file ppat.1005220.s005.TIF]

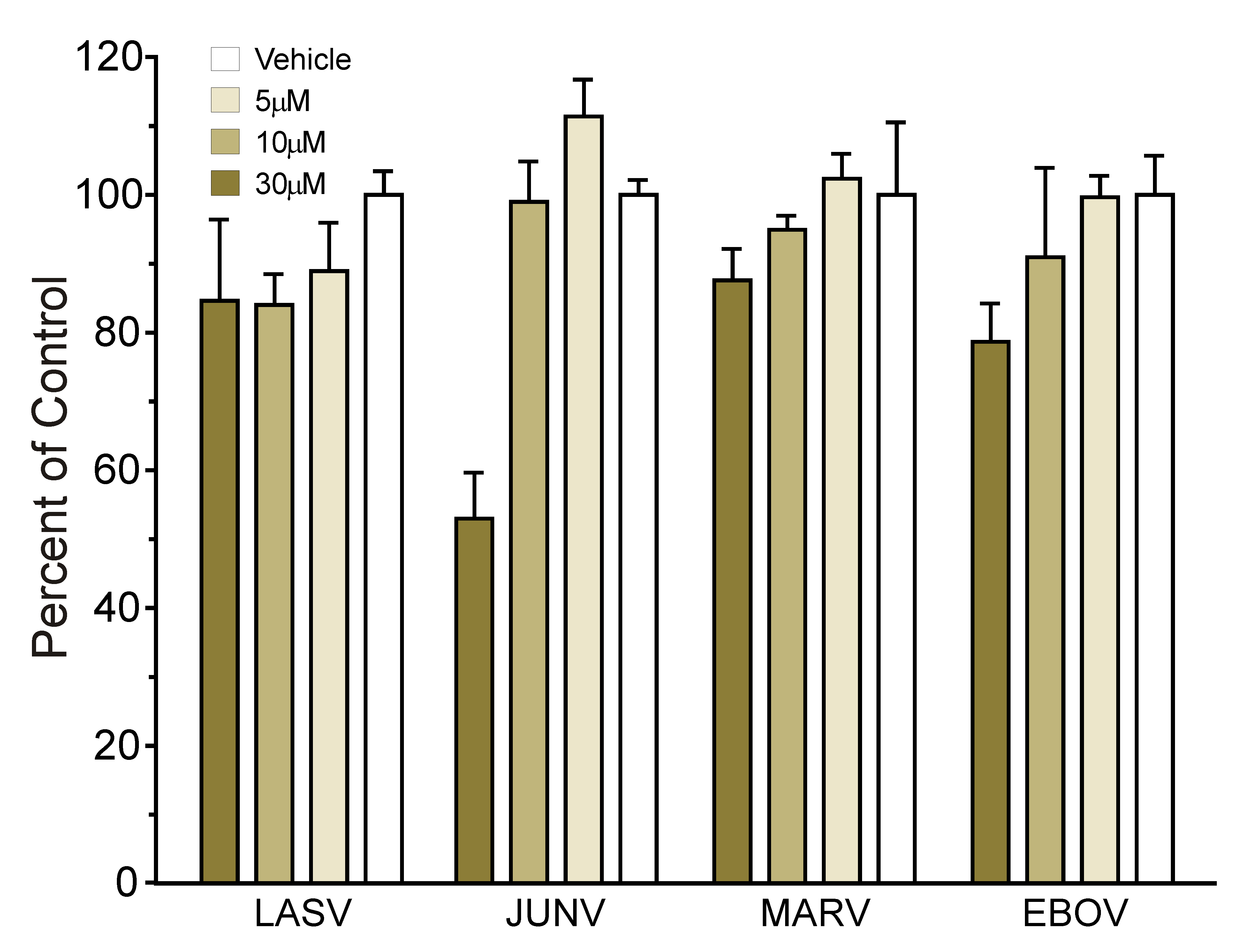

Supplement: S6 Fig — HeLa cells, seeded in 96 well plates ~24 hours prior to infection, were pretreated with Synta66 at indicated concentration or vehicle for 1 hour. Cells were infected with LASV (MOI = 0.1), JUNV (MOI = 1), MARV (MOI = 1), or EBOV (MOI = 1). One hour after infection, cells were washed and treated with fresh Synta66 or vehicle at indicated concentrations. Cells were fixed at 24 (JUNV, LASV) or 48 (MARV, EBOV) hours post infection and percent of infected cells was determined by immunofluorescence staining with virus specific antibodies. Data is expressed as the percent of vehicle (DMSO) control treated cells and error bars indicate standard error of mean (+/- SEM). (TIF) [file ppat.1005220.s006.TIF]
